# Glucose-6-phosphate dehydrogenase and its 3D structures from crystallography and electron cryo-microscopy

**DOI:** 10.1107/S2053230X24008112

**Published:** 2024-09-11

**Authors:** Stefania Hanau, John R. Helliwell

**Affiliations:** a Department of Neuroscience and Rehabilitation University of Ferrara Ferrara Italy; b Department of Chemistry University of Manchester ManchesterM13 9PL United Kingdom; Centro Nacional de Biotecnología – CSIC, Spain

**Keywords:** glucose-6-phosphate dehydrogenase, X-ray crystallography, electron cryo-microscopy, fused glucose-6-phosphate dehydrogenase–6-phosphogluconolactonase, F_420_-dependent glucose-6-phosphate dehydrogenase, human and trypanosomatid species

## Abstract

Glucose-6-phosphate dehydrogenase (G6PD) is the first enzyme in the pentose phosphate pathway. It has been extensively studied by biochemical and structural techniques. 13 X-ray crystal structures and five electron cryo-microscopy structures in the PDB are focused on in this topical review. Two F_420_-dependent glucose-6-phosphate dehydrogenase (FGD) structures are also reported. The significant differences between human and parasite G6PDs can be exploited to find selective drugs against infections such as malaria and leishmaniasis. Furthermore, G6PD is a prognostic marker in several cancer types and is also considered to be a tumour target. On the other hand, FGD is considered to be a target against *Mycobacterium tuberculosis* and possesses a high biotechnological potential in biocatalysis and bioremediation.

## Abstract 2

An investigation of different types of glucose-6-phosphate dehydrogenase (G6PD) is reported, including NAD^+^- and NADP^+^-dependent G6PDs, fused glucose-6-phosphate dehydrogenase–6-phosphogluconolactonase and bacterial F_420_-dependent G6PD. An extensive data-review tabulation of PDB entries is also provided for both X-ray crystal and electron cryo-microscopy structures.

## 1. Introduction

### 1.1. Different types of glucose-6-phosphate dehydrogenase

It is possible to recognize different types of glucose-6-phosphate dehydrogenase, namely:

Group 1: glucose-6-phosphate dehydrogenase (G6PD; EC 1.1.1.49), which catalyses the NAD^+^- or NADP^+^-dependent dehydrogenation of β-d-glucose 6-phosphate (G6P) to 6-phosphoglucono-δ-lactone.

Group 1a: this subgroup consists of G6PD fused in a bifunctional enzyme with the second enzyme of the pentose phosphate pathway (PPP), 6-phosphogluconolactonase (6PGL), and includes endoplasmic reticulum (ER) hexose-6-phosphate dehydrogenase (H6PD) and G6PDs from the parasites *Giardia* and *Plasmodium*.

Group 2: bacterial cofactor F_420_-dependent glucose-6-phosphate dehydrogenase (FGD). F_420_ is a 5-deazariboflavin that has previously been described in methanogenic archaea and claimed to be essential for antioxidant defence in mycobacteria (Eirich *et al.*, 1979 ▸; Hasan *et al.*, 2010 ▸).

### 1.2. G6PD is the first enzyme of the pentose phosphate pathway

**Figure 1:**
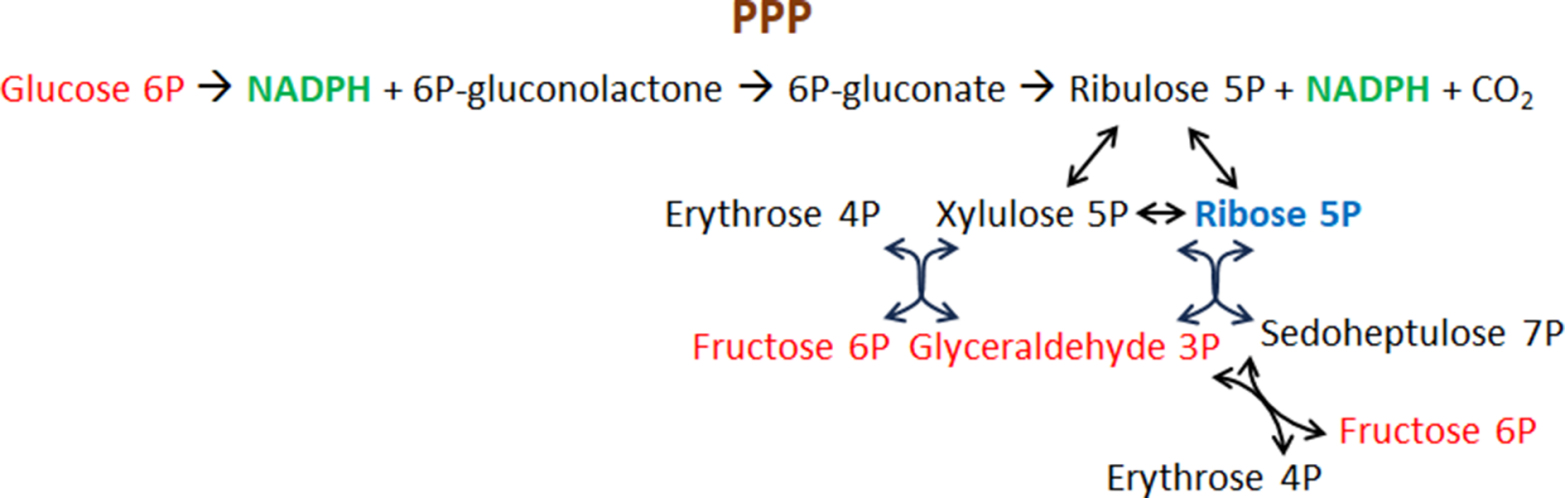
The pentose phosphate pathway (PPP). Metabolites common to glycolysis are in red.

G6PD is the first enzyme of the PPP (Fig. 1 ▸) and catalyses its rate-limiting step. It is a cytosolic enzyme that is active as a homodimer and a homotetramer (Cohen & Rosemeyer, 1969 ▸; Garcia *et al.*, 2022 ▸). In trypanosomatids it is also present in glycosomes (Gupta *et al.*, 2011 ▸).

The PPP is also named the hexose monophosphate shunt since it diverts the use of G6P in the cytosol from glycolysis to the production of both NADPH and ribose 5-phosphate (Fig. 1 ▸). The metabolic pathway divides into two phases, the oxidative PPP (OPPP) branch and the non-oxidative branch, the first of which is absent in some lower organisms such as *Cryptosporidium* (Stover *et al.*, 2011 ▸). The non-oxidative branch shares the intermediates fructose 6-phosphate and glyceraldehyde 3-phosphate with glycolysis, so that either glycolysis (or gluconeogenesis in some tissues, in particular the liver) or ribose production can be furnished according to the needs of the cell (Stincone *et al.*, 2015 ▸; García-Domínguez *et al.*, 2022 ▸). Two NADPH molecules are produced per G6P molecule in the OPPP: one is produced in the reaction catalyzed by G6PD; 6-phosphoglucono-δ-lactone is then hydrolysed by 6PGL to 6-phosphogluconate, which is then oxidatively decarboxylated to ribulose 5-phosphate by 6-phosphogluconate dehydro­genase (6PGDH), producing the second NADPH (Hanau & Helliwell, 2022 ▸).

NADPH is necessary for reductive biosynthesis, protection against oxidative stress and the function of the numerous NADPH-dependent enzymes, such as NADPH oxidases in immune cells. At the same time, it is also crucial for cancer cell metabolism and chemoresistance (Zhang *et al.*, 2021 ▸; Song *et al.*, 2022 ▸). Thus, overall, G6PD activity is regulated at different levels: firstly in the processes of transcription and RNA processing, and then at the level of the protein, such as stability, localization, post-translational modification, polymerization and allostery (Stanton, 2012 ▸; Meng *et al.*, 2022 ▸). Among the transcription factors that directly bind to a G6PD promoter, c-Myc is involved in hepatocellular carcinoma growth (Yin *et al.*, 2017 ▸), where G6PD represents a marker of poor prognosis. This is also the case for several other tumours, including lung cancer, both clear cell and papillary renal cell carcinomas, ovarian and prostate cancer, glioma, gastric and colorectal cancer (Tsouko *et al.*, 2014 ▸; Zhang *et al.*, 2020 ▸; Song *et al.*, 2022 ▸; Zeng *et al.*, 2023 ▸; Thakor *et al.*, 2024 ▸). G6PD has also been found to be related to angiogenesis and distant metastasis (Zeng *et al.*, 2023 ▸). In breast cancer Nrf2 was shown to be the transcription factor that is involved (Zhang *et al.*, 2019 ▸), while in glioblastoma and renal cell carcinoma it is STAT3 (Song *et al.*, 2022 ▸). The G6PD gene also contains vitamin D and sterol regulatory element-binding protein response elements and p53 family protein response elements. Furthermore, several other transcription factors have been reported to bind to the G6PD promoter (Meng *et al.*, 2022 ▸).

G6PD deficiency is the most common human enzymopathy, affecting 500 million people worldwide. This arises due to mutation of the gene, which is located in the X chromosome. The mutation affects above all the red blood cells, causing hemolytic anaemia of different grades, which can be triggered by exposure to oxidative stress and was classified in 1989 by the World Health Organization (WHO) into classes I–IV, with the first being the most severe (WHO Working Group, 1989 ▸). This increased sensitivity to oxidative damage can manifest after the consumption of fava beans (favism), other foods, medications, especially antimalarial drugs, during infections and under particular environmental conditions (https://www.g6pd.org/en/G6PDDeficiency/SafeUnsafe/DaEvitare_ISS-it). A new WHO classification foresees four classes, A, B, C and U, where variants formerly in classes II and III are merged into the single class B (Luzzatto *et al.*, 2024 ▸). On the other hand, deficient individuals and female carriers of deficient alleles are provided with beneficial effects against malaria, which is the reason why the frequency of G6PD deficiency is so high in those geographical regions with a high incidence of malaria (Mason *et al.*, 2007 ▸; Nkhoma *et al.*, 2009 ▸). G6PD deficiency has also been related to a longevity pattern (Schwartz & Pashko, 2004 ▸).

## 2. Crystallographic and electron cryo-microscopy (cryoEM) structures

In Table 1 ▸ we cite many of the available crystallographic structures and their PDB codes and report various details. We selected these structures based on their representativeness. The table includes a column in which peaks that are unmodelled in the PDB entry have been scrutinized using the *Coot* molecular-graphics visualization system (Emsley *et al.*, 2010 ▸). Table 1 ▸ also includes a column in which the PDB validation reports are assessed by their clashscore. Specific comments of interest based on the PDB reports and an evaluation of the structure factors are also provided in this column. In Table 2 ▸ we cite cryo-EM structures and include a similar validation evaluation.

### 2.1. The G6PD subunit

**Figure 2:**
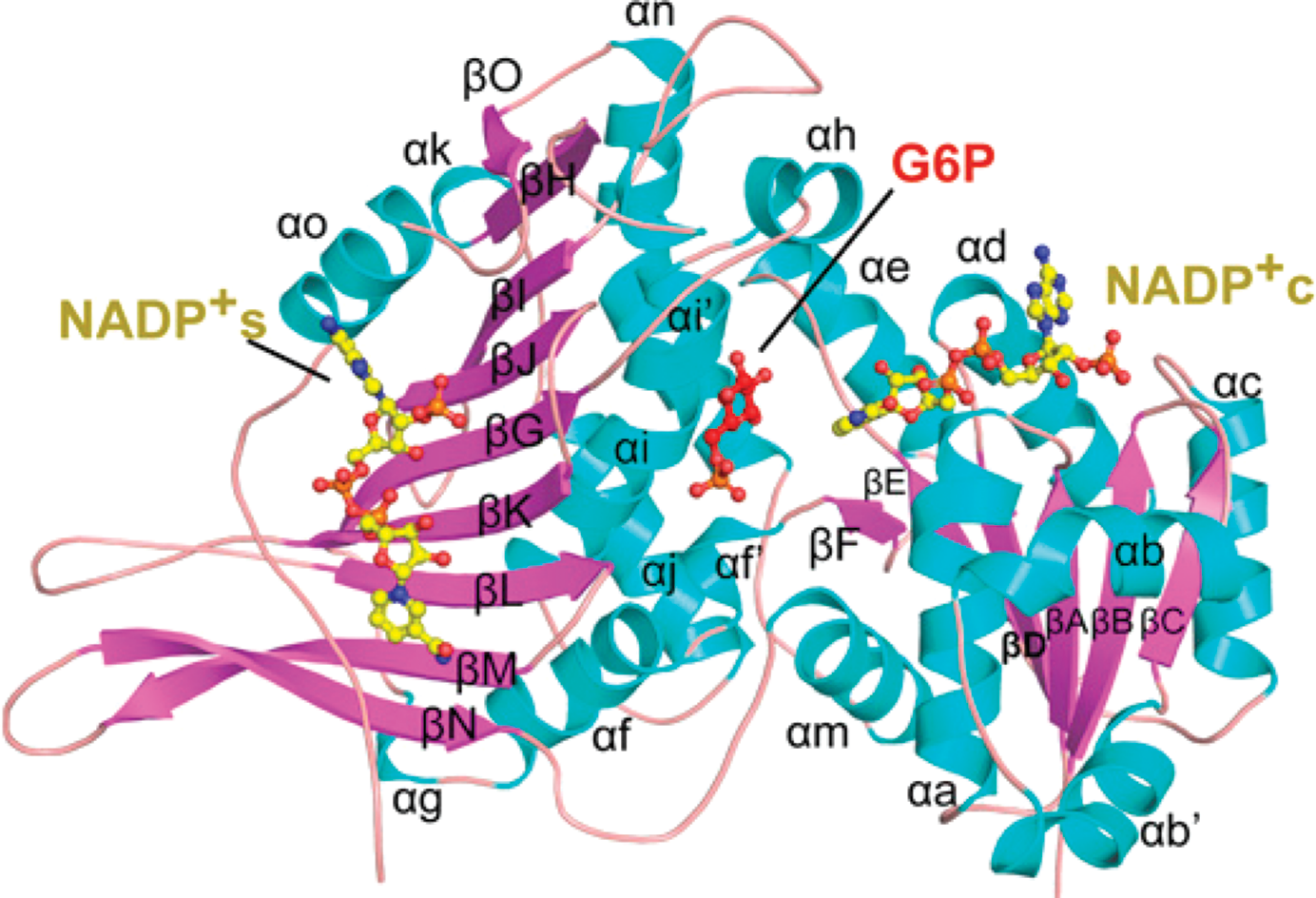
Ribbon diagram of the human G6PD subunit in complex with structural NADP (NADP^+^s), catalytic NADP (NADP^+^c) and G6P (PDB entry 7sni; reproduced from Wei *et al.*, 2022 ▸).

**Figure 3:**
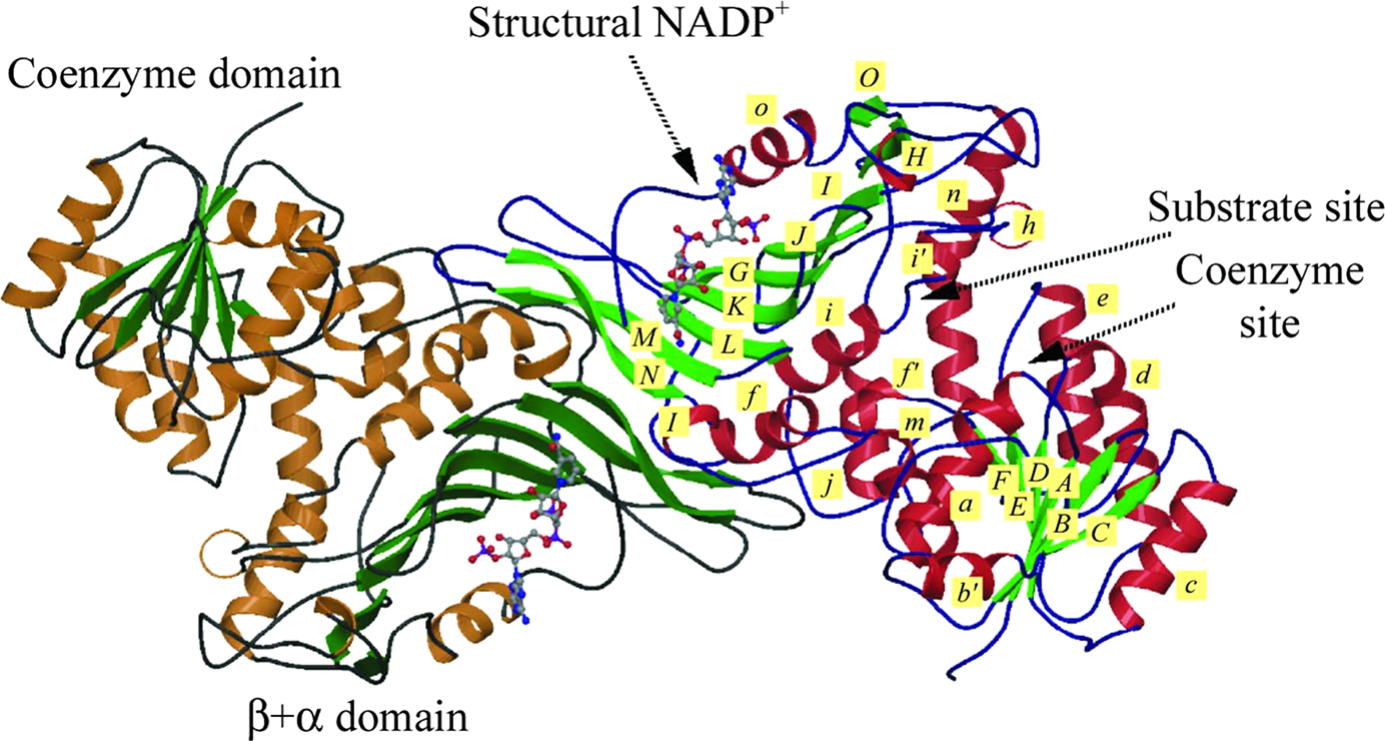
The human G6PD_Canton_ (R459L) dimer with structural NADP^+^ bound (PDB entry 1qki; reproduced from Kotaka *et al.*, 2005 ▸).

The molecular weight of the human G6PD monomer is 59 kDa (Au *et al.*, 2000 ▸). The first G6PD crystal structures, that from *Leuconostoc mesenteroides* (LM; PDB entry 1dpg) and the human form (PDB entry 1qki), both showed an N-terminal β–α–β ‘Rossmann-fold’ coenzyme-binding domain and a β+α domain dominated by a curved nine-stranded antiparallel β-sheet (Fig. 2 ▸; Rowland *et al.*, 1994 ▸; Au *et al.*, 2000 ▸). In the amino-acid numbering system used in this paper the N-terminal methionine is designated ‘1’. The first crystal structure of human G6PD was that of the class II mutant Canton R459L, which is one of the most common Chinese variants; it has only 18% of normal G6PD activity due to its decreased stability and has a higher *K*_m_ for G6P in particular (Fig. 3 ▸; Au *et al.*, 2000 ▸; Hwang *et al.*, 2018 ▸). While no cysteine is present in the LM enzyme, there are seven cysteines in the human enzyme, with a disulfide bridge between Cys13 and Cys446, which most probably orders the mobile N-terminal segment (Au *et al.*, 2000 ▸). After these structures, crystal structures of a deletion mutant ΔG6PD (*i.e.* without 25 N-terminal residues which were poorly ordered) were solved as binary complexes with G6P (PDB entry 2bhl) and NADP^+^ (PDB entry 2bh9) (Kotaka *et al.*, 2005 ▸).

**Figure 4:**
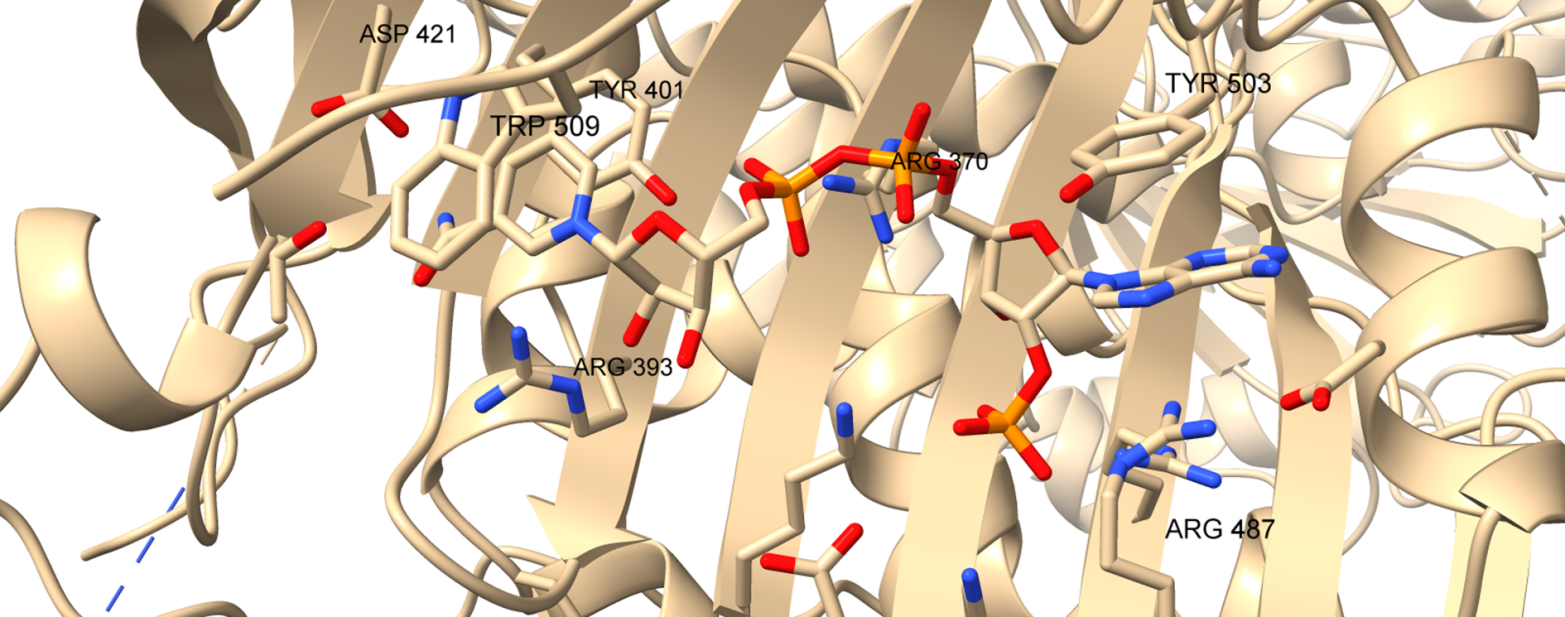
Structural NADP binding site in human G6PD_Canton_ (R459L) (PDB entry 1qki). This figure was prepared in *UCSF ChimeraX* (Meng *et al.*, 2023 ▸; https://www.rbvi.ucsf.edu/chimerax).

In all higher organisms an additional allosteric binding site for NADP^+^ between the β-sheet and the C-terminus, proximal to the dimer interface, supports the binding of a second NADP^+^, called the ‘structural’ NADP^+^ (NADP^+^s), which is not present in the LM enzyme (Fig. 2 ▸). NADP^+^s structurally stabilizes the enzyme, promotes oligomer assembly and regulates G6P binding and catalysis (De Flora *et al.*, 1974 ▸; Au *et al.*, 2000 ▸; Wang *et al.*, 2008 ▸; Garcia *et al.*, 2022 ▸; Wei *et al.*, 2022 ▸). Thus, when the cytosolic ratio [NADPH]/[NADP^+^] decreases, NADP^+^ binding to the regulatory site of G6PD should allow the activation of G6PD, which is thought to work at low efficiency under normal cell conditions while being necessary during oxidative stress (Filosa *et al.*, 2003 ▸). Although a second dinucleotide fingerprint is present in G6PDH sequences, this sequence does not appear to be involved in NADP^+^s binding in the structure of the human enzyme, and contacts are all made to side-chain atoms of one subunit (Fig. 3 ▸; PDB entries 1qki and 6e08; Au *et al.*, 2000 ▸). NADP^+^s is 77% buried in the protein at a positively charged binding site with many arginine and lysine residues, at a distance of 7 Å from residues of the second subunit (Fig. 4 ▸). Its nicotinamide amide binds to Asp421, which is at the centre of the dimer interface. The aromatic rings of the adenine and nicotinamide form π–π interactions, with the adenine being between Tyr503 and Arg487, while the nicotinamide is between Trp509 and Tyr401 (Fig. 4 ▸; Au *et al.*, 2000 ▸; Kotaka *et al.*, 2005 ▸).

Phosphorylation of the last residue by the Src-family kinase Fyn in red blood cells has been shown to activate G6PD (Mattè *et al.*, 2020 ▸). Glu416, Glu417 and Glu419 can attract the positive charge on the oxidized nicotinamide ring. Hence, NADPH is not well bound at this site (Au *et al.*, 2000 ▸; Kotaka *et al.*, 2005 ▸). NADP^+^s is present in crystals of the human enzyme (containing tetramers) even if the coenzyme is not present in the protein solution prior to crystallization (Kotaka *et al.*, 2005 ▸; Hwang *et al.*, 2018 ▸). The *K*_d_ for NADP^+^s is 37 n*M*, which is 200-fold lower than that for the ‘catalytic’ NADP^+^ (NADP^+^c; Wang *et al.*, 2008 ▸). However, it is not present in the binary complex with G6P, in which the C-terminus is disordered (Kotaka *et al.*, 2005 ▸). It is not present in cryo-electron microscopy (cryo-EM) structures of G6PD (tetramer; PDB entry 7sng), in which the C-terminus is also disordered (Wei *et al.*, 2022 ▸). It was in fact shown that dimeric G6PD without NADP^+^s is active, although it is much less stable (Wang *et al.*, 2008 ▸). Also, NADP^+^s can be reduced in the presence of G6P after migrating to the catalytic site (Kotaka *et al.*, 2005 ▸; Wang *et al.*, 2008 ▸).

**Figure 5:**
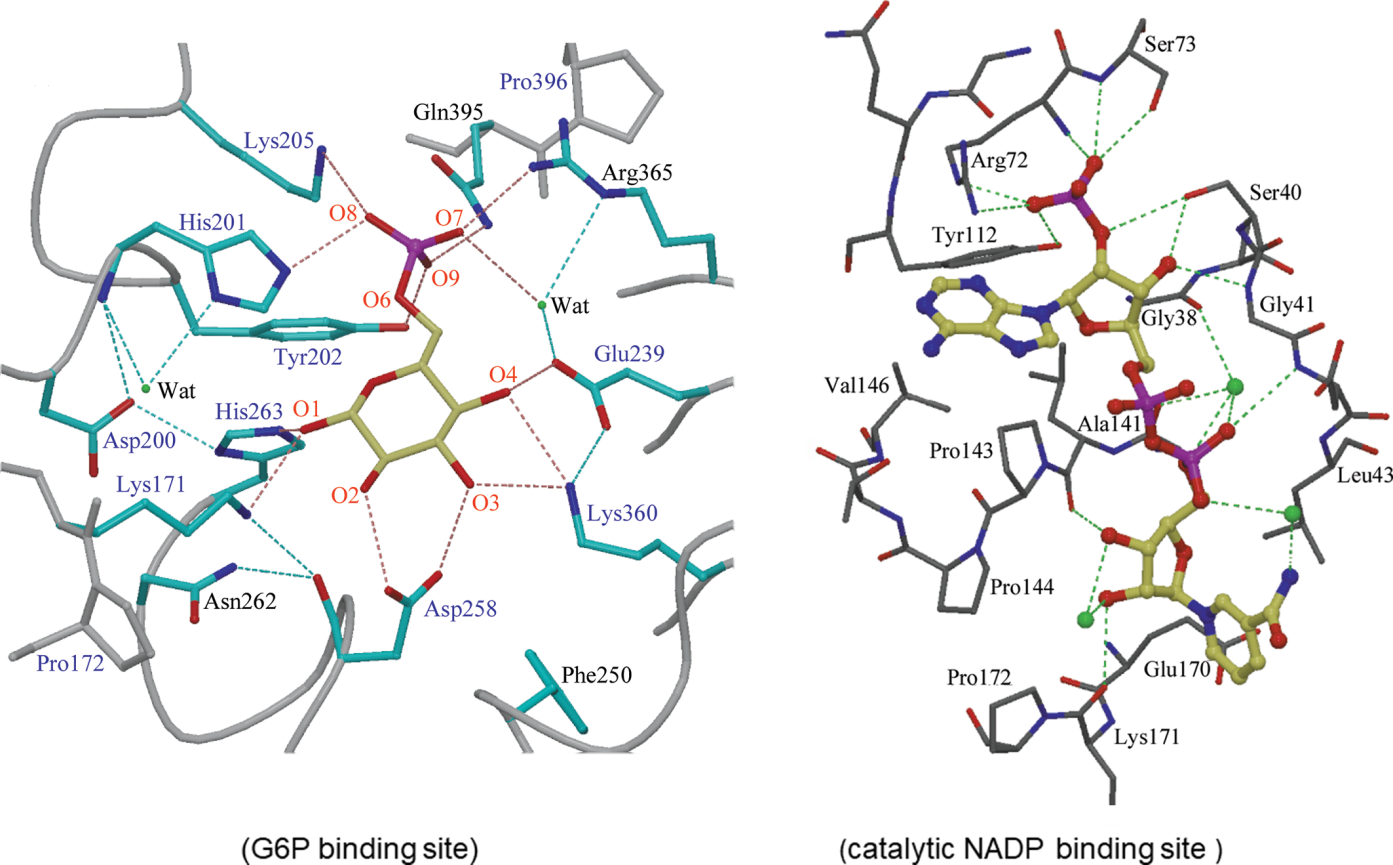
The human G6PD–G6P complex (left; PDB entry 2bhl) and G6PD–catalytic NADP^+^ complex (right; PDB entry 2bh9). Reproduced from Kotaka *et al.* (2005 ▸).

**Figure 6:**
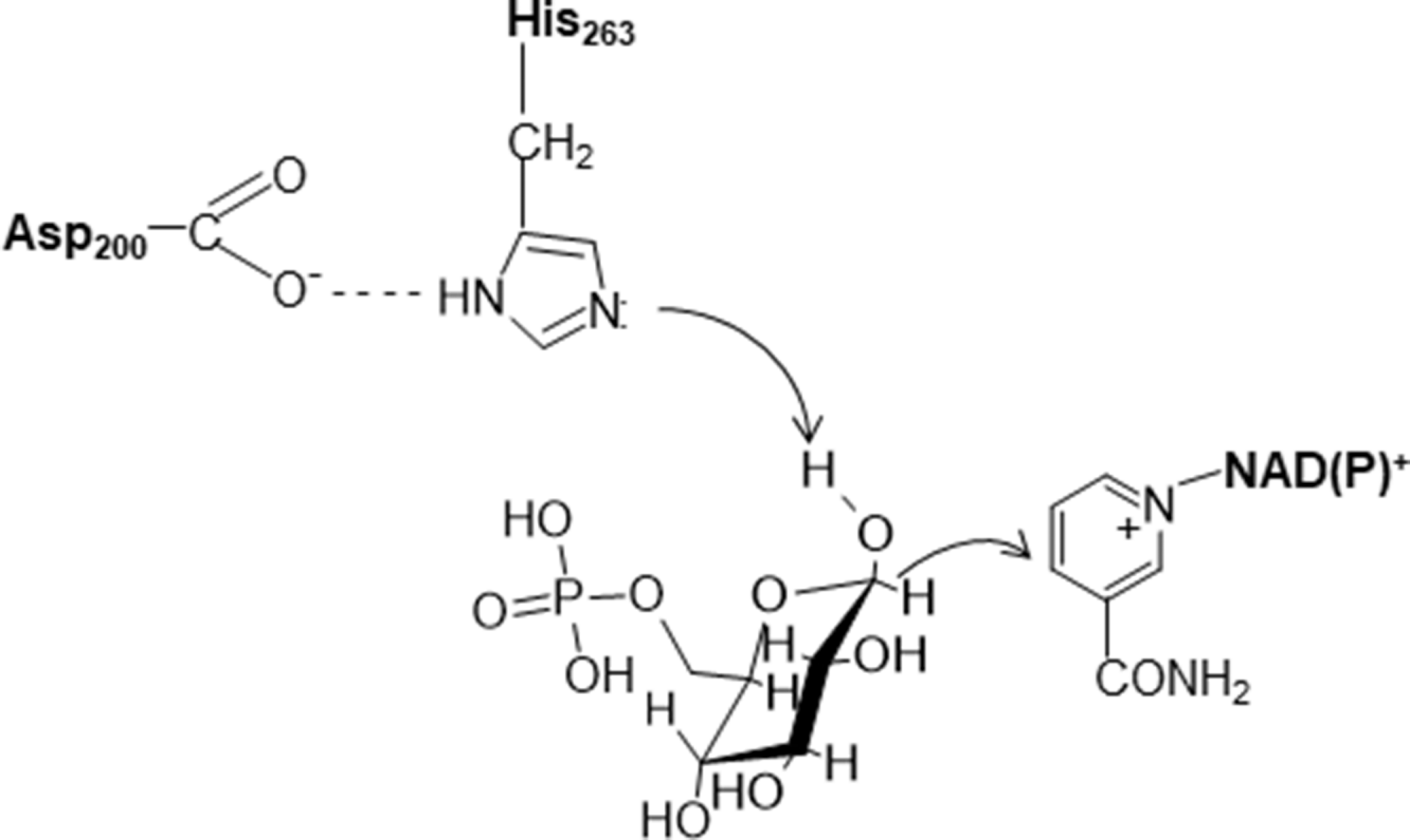
The His–Asp catalytic dyad of human G6PD.

G6P binds in the pocket between the N- and the C-terminal subdomains proximal to NADP^+^c (Fig. 2 ▸) at a highly conserved nine-residue region in helix αf′, with Asp200 involved in catalysis and His201, Tyr202 and Lys205 involved in substrate phosphate binding (Fig. 5 ▸, left) (Au *et al.*, 2000 ▸; Cosgrove *et al.*, 2000 ▸; Kotaka *et al.*, 2005 ▸; Mason *et al.*, 2007 ▸; Wei *et al.*, 2022 ▸). His263 is the general base, which is hydrogen-bonded to Asp200, which together form a catalytic dyad as found in other enzymes. The Asp200 carboxylate stabilizes the positive charge of the histidine after proton abstraction from the C1 hydroxyl of G6P, allowing transfer of the C1 hydride to the nicotinamide ring of the coenzyme (Fig. 6 ▸; Cosgrove *et al.*, 2000 ▸). Some differences between the LM and human G6PDs are present in the region of the binding of 6PG to elements near the mixed β-sheet, such as the positions of Asp258 from helix αi, Lys360 from a loop of the β-sheet and of human Gln395 binding to the phosphate (Fig. 5 ▸, left; Kotaka *et al.*, 2005 ▸; Wei *et al.*, 2022 ▸). This Gln is near Arg393 in the β-sheet, interacting with the O atom of the amide of NADP^+^s (Fig. 4 ▸).

In the polypeptide loop between the βJ and βK strands, Arg365 binds the phosphate of G6P, while Lys366 at the beginning of the βK strand interacts with the 2′-phosphate of NADP^+^s (Figs. 2 ▸, 3 ▸ and 5 ▸, left). Also, other residues in this region interact by means of bound water molecules, allowing communication between NADP^+^s and the substrate-binding sites (for instance Asn363). Many class I variants only have mutations at these positions (Kotaka *et al.*, 2005 ▸; Mason *et al.*, 2007 ▸; Hernández-Ochoa *et al.*, 2023 ▸).

The dinucleotide-binding fingerprint G*xx*GDL*x* encompasses residues 38–44 in the βA–αA turn, with the main-chain amino groups of Gly41 and Asp42 hydrogen-bonded to the O atoms of the adenine ribose 3′-hydroxyl and the bisphos­phate (Fig. 5 ▸, right). Arg72 (Arg46 in the LM enzyme) is proximal to the 2′-phosphate (Fig. 5 ▸, right); however, there are differences in the catalytic coenzyme binding site between the human and LM enzymes that are related to the potential of the LM enzyme, but not the human enzyme, to use both NAD^+^ and NADP^+^. Among these, LM G6PD has a more hydrophobic environment for the adenine ring (Rowland *et al.*, 1994 ▸; Levy, 1979 ▸; Kotaka *et al.*, 2005 ▸). The hinge angle between the two domains of LM G6PD can vary, allowing three different shapes: a closed structure binding G6P, a half-open structure with NAD^+^ bound and an open form that tightly binds NADP^+^ (Naylor *et al.*, 2001 ▸). The NADP^+^ reaction is an ordered sequential reaction, with G6P binding after NADP^+^. Fluorescence studies indicate that upon G6P binding there is an open-to-closed conformational change (Naylor *et al.*, 2001 ▸; Haghighi & Levy, 1982 ▸). In both NADP^+^- and NAD^+^-bound structures the nicotinamide ribose makes a hydrogen bond to Lys148 (Lys171 in the human enzyme; Fig. 5 ▸, right) of the conserved EKP*x*G sequence. The *cis*–*trans* isomerization of the proline in this region in helix αe is critical in allowing the lysine to interact with both O1 of G6P and the 3′-hydroxyl of the nicotinamide ribose for the correct positioning of nicotinamide (Fig. 5 ▸; Bautista *et al.*, 1995 ▸; Kotaka *et al.*, 2005 ▸). A P172S mutation is found in the class I Volendam G6PD, which leads to chronic nonspherocytic hemolytic anaemia (CNSHA) and also seriously affects G6PD activity in leucocytes (Roos *et al.*, 1999 ▸). In mammalian cells the conserved Ser84 was identified to be O-GlcNAcylated in a small percentage of G6PD; glycosylation increased under hypoxia or other conditions and has been found to significantly increase in lung and oesophageal cancer tissue compared with adjacent normal tissue (Rao *et al.*, 2015 ▸; Su *et al.*, 2021 ▸). When induced by means of the overexpression of O-GlcNAc transferase, glycosylation resulted in a higher NADP^+^ binding affinity (Rao *et al.*, 2015 ▸). Ionizing radiation-induced Thr145 phosphorylation by casein kinase 2 in human epithelial cells also increases NADP^+^ binding affinity; hence, the G6PD activity increases (Hao *et al.*, 2023 ▸).

**Figure 7:**
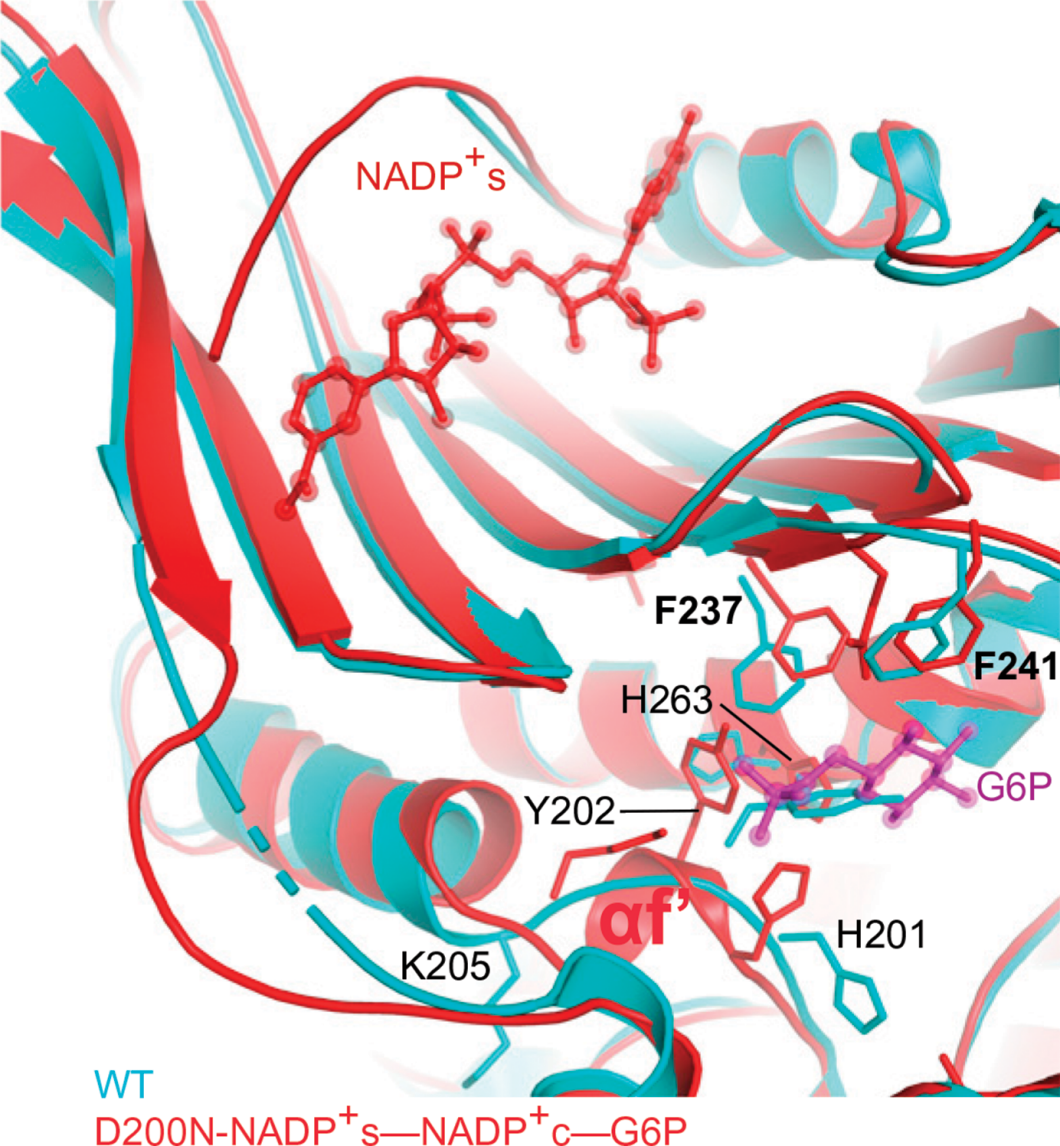
Superposition of G6PD WT and G6PD D200N–NADP^+^s–G6P suggesting NADP^+^s-mediated reorientation of Phe237 and Phe241, repositioning His201, Tyr202 and Lys205 (forming helix αf′), and of the catalytic His263 (reproduced from Wei *et al.*, 2022 ▸).

Comparisons of the cryo-EM structure of the D200N G6PD mutant bound to all of the ligands (PDB entry 7sni), the crystal structure with NADPs bound (PDB entry 1qki) and the cryo-EM structure of wild-type G6PD (G6PD WT; PDB entry 7sng) are informative. These suggest that a structural ordering of the subunit C-terminus in the presence of allo­steric NADP^+^s involves movement of Phe237 and Phe241 from the β-sheet and flips of His201, Tyr202 and Lys205, which would be in a position that is incompatible with G6P binding in the apo­enzyme. At the NADP^+^s binding site the loop 201–204 would form helix αf′, and His263 moves from a catalytically incompetent location to a competent location (Wei *et al.*, 2022 ▸; Fig. 7 ▸). Tyr112 has been reported to be phosphorylated by tyrosine kinase c-Src in colorectal cancer, causing increases in 6PG affinity and enzyme activity (Ma *et al.*, 2021 ▸). In contrast, in breast cancer cells (MCF7) the Src protein was found to decrease G6PD activity by phosphorylating Tyr249 and Tyr322, which are spatially close to the G6P binding pocket, and this is reverted in the presence of 20 m*M* lactic acid, mimicking the metabolic microenvironment of the solid tumour (Sun *et al.*, 2023 ▸).

### 2.2. The active forms of G6PD are both as a dimer and as a tetramer

**Figure 8:**
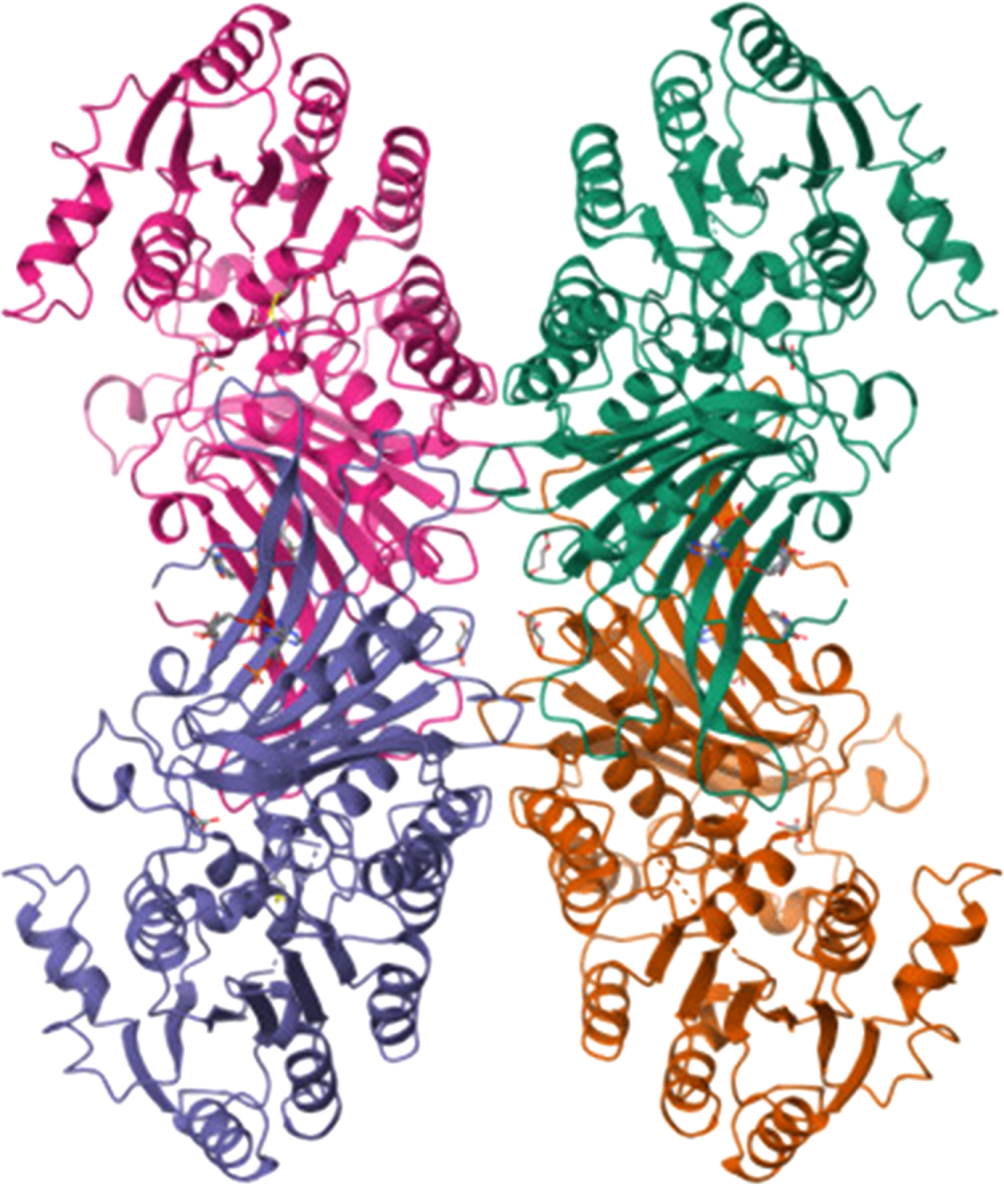
The human G6PD tetramer (Au *et al.*, 2000 ▸; PDB entry 1qki).

While LM G6PD is a dimer, the human enzyme in solution is in a monomer–dimer–tetramer equilibrium, where the monomer is not active and is induced by NADPH in a regulatory way (Bonsignore *et al.*, 1971 ▸). The dimer–tetramer equilibrium is shifted towards the dimer by one of the following: a pH higher than 8, a high ionic strength, enzyme dilution or the presence of G6P (Cohen & Rosemeyer, 1969 ▸; Cancedda *et al.*, 1973 ▸; Horikoshi *et al.*, 2021 ▸). In contrast, NADP^+^ and certain metal ions favour the tetramer (Kirkman & Hendrickson, 1962 ▸; Wang *et al.*, 2008 ▸), which has been shown to be more active and more stable than dimeric G6PD (Ranzani & Cordeiro, 2017 ▸; Garcia *et al.*, 2022 ▸). The crystallized human G6PD is a tetramer consisting of a side-by-side dimer of dimers (Au *et al.*, 2000 ▸; Fig. 8 ▸), while high-resolution cryo-electron microscopy structures show a mixture of dimers and tetramers, with an overall structure like that in the crystal (Wei *et al.*, 2022 ▸).

57 amino-acid residues are involved in the dimer interface of human G6PD, while there are 48 in the LM enzyme, although in both approximatively half are hydrophobic and the gross structure of the interface is conserved. Hydrogen bonds and salt bridges are present, and the two βN strands of the β-sheets are associated in both cases (Fig. 3 ▸; Au *et al.*, 2000 ▸; Rowland *et al.*, 1994 ▸). In the human enzyme dimer, the longer C-terminal tail is flexible unless NADP^+^s is bound in the positively charged crevice between the β-sheet and the C-terminus (Kotaka *et al.*, 2005 ▸). Lys407 is a completely conserved residue in the interface. Close by, Lys403 is an NADP^+^s-bound conserved residue that is found to be acetylated in immortalized cells and mouse embryonic fibroblasts. This acetylation hinders dimerization, causing enzyme inactivation, while NAD^+^-dependent SIRT2 can directly reactivate G6PD by deacetylation of Lys403 in response to oxidative stress (Wang *et al.*, 2019 ▸). In agreement with this, most of the G6PD variants that cause deficiencies of classes I and II have mutations in the dimer interface region and at the NADP^+^s binding site, thereby showing decreased activity and stability (Au *et al.*, 2000 ▸; Mason *et al.*, 2007 ▸; Gomes-Manzo *et al.*, 2017 ▸; Luzzatto *et al.*, 2020 ▸; Chandran *et al.*, 2024 ▸). In certain mutants, enzyme activity recovers on increasing the NADP^+^ concentration and stability increases when it is present (Beutler *et al.*, 1991 ▸; Hernández-Ochoa *et al.*, 2023 ▸). There is ongoing active research in finding molecules that activate and also stabilize G6PD mutants by promoting dimer formation (Hwang *et al.*, 2018 ▸; Raub *et al.*, 2019 ▸). G6PD^A−^ (V68M and N126D), a class III double variant and is also the most common African variant (WHO Working Group, 1989 ▸; Nkhoma *et al.*, 2009 ▸), has significantly improved activity when the dimers are stabilized (Garcia *et al.*, 2022 ▸). Sirtuin 5 can activate G6PD by deglutarylating it, although the residue involved is not known to date (Zhou *et al.*, 2016 ▸). Lactylation, specifically of Lys45, was also found to inhibit G6PD, and the oncoprotein E6 of human papilloma virus HPV16 causing cervical cancer can revert it, thus activating the PPP (Meng *et al.*, 2024 ▸). Lys403, together with Lys366, was also shown to be the main ubiquitination site of G6PD (Wang *et al.*, 2019 ▸).

The residues involved in tetramerization are mostly charged, coming from the junction αi–αj, αj and the βI–βJ and βK–βL loops. They form few salt bridges, thus explaining the sensitivity of the dimer–tetramer equilibrium to pH or ionic strength (Au *et al.*, 2000 ▸; Kotaka *et al.*, 2005 ▸). Only a quarter of the surface area (706 Å^2^ from the two dimers) is buried compared with the monomer surface covered in dimer formation (Fig. 8 ▸; Au *et al.*, 2000 ▸; Kotaka *et al.*, 2005 ▸). Nevertheless, a spatial scan statistic obtained by analysing pathogenic and benign G6PD variants suggested that disruption of tetramerization decreases the enzyme activity, and is frequently found in class II variants (Cunningham & Mochly Rosen, 2017 ▸). Also, several class I mutants and the K403Q mutant were shown to be dysfunctional dimers that are less prone to dissociation and are unable to form tetramers (Garcia *et al.*, 2022 ▸). A change in the NADPs binding site like that in the K403Q mutant (PDB entry 7sei) caused allosteric modification in the substrate-binding site, with helix αf moving in a way that hinders G6P binding (Garcia *et al.*, 2022 ▸). At the same time, mutations at the dimer interface (F216L) or the tetramer interface (E274K and K275N) induce an allosteric loss of NADPs binding and a dysfunctional dimer. In both cases tetramerization is missing (Garcia *et al.*, 2022 ▸). Induced tetramerization by the introduction of cysteines and disulfide bonds increases the activity and stability of both the WT and mutants such as G6PD_Canton_ and G6PD_Med_ (S188F) (Garcia *et al.*, 2022 ▸). The latter is a class II variant that is most prevalent in the Middle East and is highly prevalent in India (Mason *et al.*, 2007 ▸; Sukumar *et al.*, 2004 ▸).

Horikoshi and coworkers have solved structures of class I mutants: V394L (a residue in the β–α interaction site), F381L (a residue in the dimer interface), P396L and R393H (residues in the NADPs site) (Fig. 4 ▸). These have impaired NADP^+^ binding and are not able to dissociate into monomers or to form tetramers (Garcia *et al.*, 2022 ▸). In these structures the βM–βN strands are disordered, causing a shift of the αf helix into the G6P-binding site and its occlusion by the αf′ helix (Fig. 7 ▸), highlighting the long-distance communication between the NADPs and the G6P binding sites (Horikoshi *et al.*, 2021 ▸).

In cancer cells, phosphorylation of Thr406 and Thr466 by Polo-like kinase 1 in the regulating cell cycle has been shown to affect G6PD dimerization and activity (Ma *et al.*, 2017 ▸), while protein kinase A inhibits G6PD by serine phosphorylation (Xu *et al.*, 2005 ▸). Conversely, phosphorylation of Ser40 (Fig. 5 ▸, right) by NF-κB-inducing kinase in CD8^+^ effector T cells activates the enzyme, the glycolytic enzyme hexokinase 2 and antitumor immunity (Gu *et al.*, 2021 ▸).

## 3. Reported ligands of G6PD

**Figure 9:**
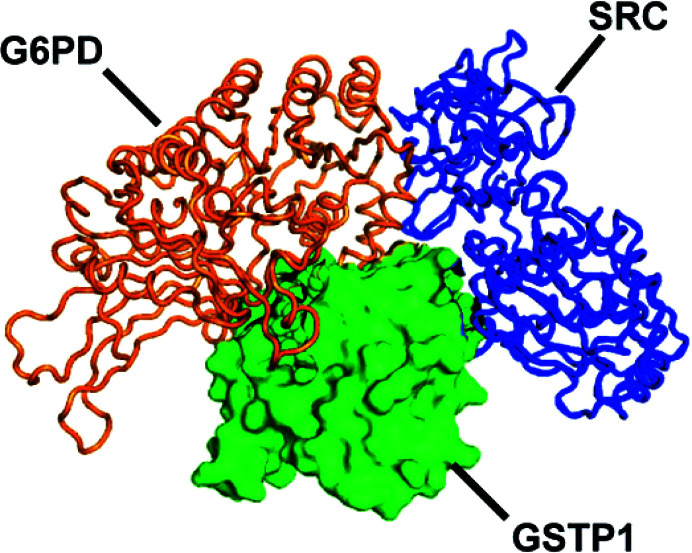
A modelled spatial arrangement of the GSTP1–SRC–G6PD complex. Green, GSTP1 (PDB entry 3gus); blue, SRC (PDB entry 2h8h); orange, G6PD (PDB entry 2bhl) [reproduced from Sun *et al.* (2023 ▸) according to https://creativecommons.org/licenses/by/4.0/].

As well as the detailed protein assembly described thus far, it is interesting to explore the ways in which G6PD can bind to each of the large number of proteins that have so far been reported to associate with it (Malhotra *et al.*, 2021 ▸; He *et al.*, 2022 ▸). Enzymes of the PPP, including G6PD, can form a supramolecular complex that can increase the shunt efficiency by substrate channelling (Huang *et al.*, 2005 ▸). Furthermore, growth factors stimulate G6PD phosphorylation, such as VEGF activating the enzyme by Tyr428 and Tyr507 Akt phosphorylation, which allows translocation of G6PD to the cell membrane (Pan *et al.*, 2009 ▸). Acetylation and other post-translational modifications have also been reported in the previous sections. Also, under glucose deprivation glutathione *S*-transferase P1 (GSTP1) has been shown to *S*-glutathionylate G6PD in MCF7 cells, while this modification is reduced in the presence of lactic acidosis, supporting an increase in both G6PD activity and cell proliferation (Sun *et al.*, 2023 ▸). Moreover, GSTP1 has been proposed as a lactic acid sensor since this metabolite would hamper the formation of a tripartite complex of GSTP1, G6PD and Src (Fig. 9 ▸; Sun *et al.*, 2023 ▸).

Giving the low basal activity and the high cytosolic NADPH/NADP^+^ ratio, G6PD binding to activating proteins was suggested a long time ago (Eggleston & Krebs, 1974 ▸; Barcia-Vieitez & Ramos-Martinez, 2014 ▸). Binding to Hsp27 activates G6PD (Cosentino *et al.*, 2011 ▸), and SUMOylation has also been shown to stabilize G6PD after its deacetylation by SIRT2 (Ni *et al.*, 2021 ▸). Conversely, HSPA8/HSC70 binds to G6PD, eliminating it by a chaperone-mediated autophagy process (Deng *et al.*, 2023 ▸), while monomeric cytoplasmic p53 binds to G6PD and prevents the formation of an active dimer (Jiang *et al.*, 2011 ▸). The enzyme hepatic aldolase B (AldoB) potentiates p53 inhibition through an AldoB–G6PD–p53 protein complex (Li *et al.*, 2020 ▸). The interaction of *Leishmania donovani* G6PD with trypanothione reductase has been shown to potentiate the peculiar and fundamental parasite thiol antioxidant machinery (Ghosh *et al.*, 2017 ▸). Finally, G6PD has been shown to support metastasis by upregulating the redox-balance capacity, since its active dimer and tetramer can bind and activate NAD kinase I (Zhang *et al.*, 2021 ▸, 2022 ▸).

**Figure 10:**
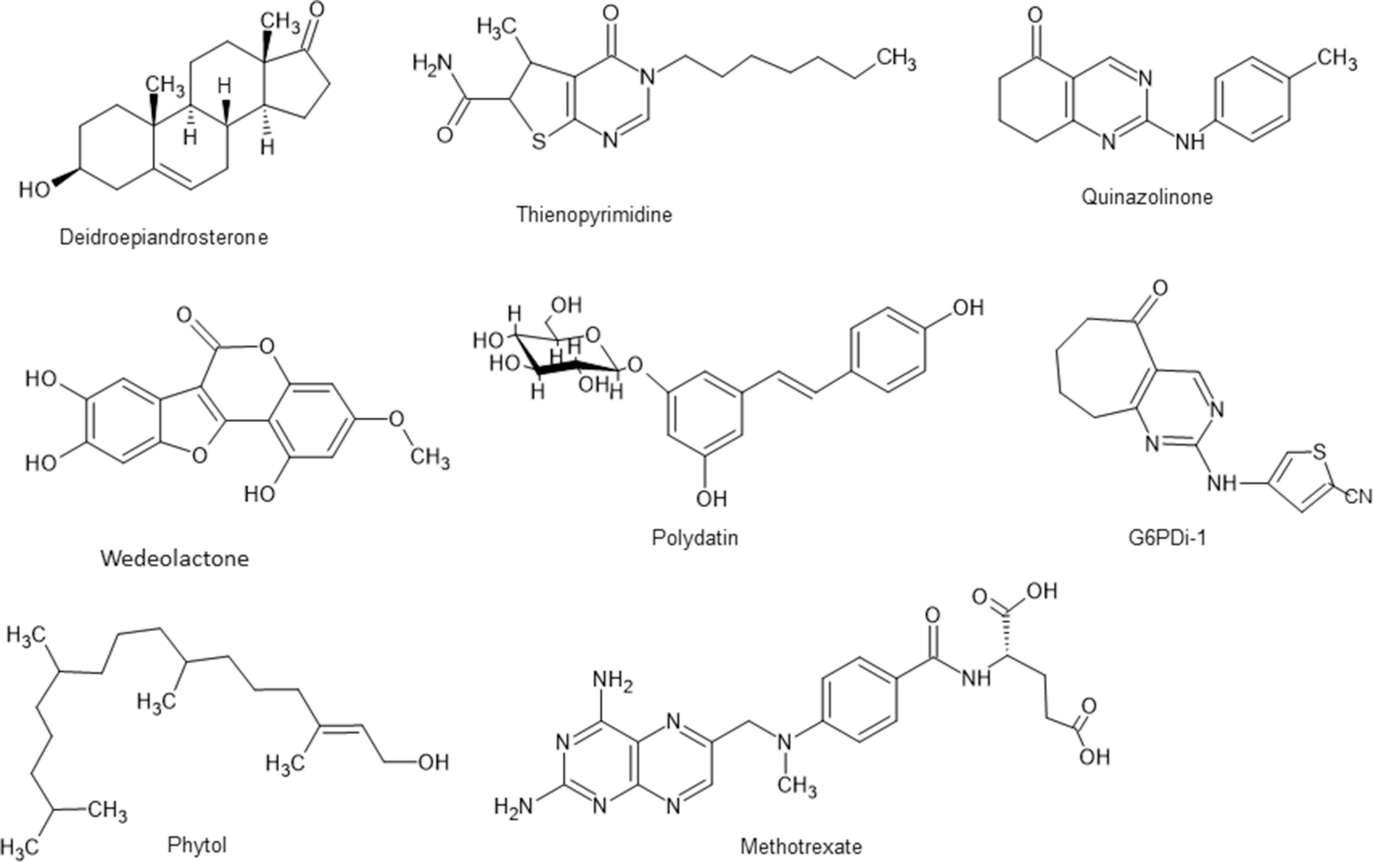
Representative G6PD inhibitors.

In Fig. 10 ▸ only a few of the compounds that inhibit the enzyme are shown. The steroid dehydroepiandrosterone (DHEA) and its derivatives are strong uncompetitive inhibitors of mammalian and parasite G6PD with respect to both substrate and coenzyme, indicating that they only bind to the ternary complex (Marks & Banks, 1960 ▸; Gordon *et al.*, 1995 ▸). Molecular docking has predicted binding in the active site (Zhao *et al.*, 2014 ▸). Quinazolinone and thienopyrimidine derivatives have been shown to compete with steroid inhibitors and some were selective for the *Trypanosoma cruzi* enzyme compared with the human enzyme (Mercaldi *et al.*, 2014 ▸).

Wedeolactone, a noncompetitive reversible G6PD inhibitor with a *K*_d_ of 3.6 µ*M*, was found by high-throughput screening (Luo *et al.*, 2021 ▸). Polydatin is another natural compound (resveratrol glucoside) that has been shown to inhibit G6PD (Mele *et al.*, 2018 ▸). Using *in silico* molecular docking, phytol and methotrexate have been predicted to have a good binding affinity (Thakor *et al.*, 2017 ▸), while G6PDi-1, which was developed to inhibit G6PD in immune cells, showed a half-maximal G6PD inhibition of 100 n*M* in astrocyte cultures (Watermann *et al.*, 2023 ▸).

## 4. G6PD in trypanosomatids

G6PD is considered to be a target to kill these parasites since it is highly important in defending them from the immune system of the host (Opperdoes & Michels, 2001 ▸; Gupta *et al.*, 2011 ▸; Kovářová & Barrett, 2016 ▸). Compared with the human enzyme it possesses an additional N-terminal domain. This domain also differs among the three species *T. cruzi*, *T. brucei* and *Leishmania* (Igoillo-Esteve & Cazzulo, 2006 ▸; Gupta *et al.*, 2011 ▸).

No structural NADP^+^ has been reported nor a dimer–tetramer equilibrium, even in the presence of ligands or Mg^2+^ or by varying the ionic strength, in either *T. cruzi* and *L. donovani*, and in the latter even over the pH range 4.8–7.8 (Ortíz *et al.*, 2019 ▸; Berneburg, Rahlfs *et al.*, 2022 ▸). The *T. cruzi* G6PD tetramer is particularly stable, having more salt bridges than the human tetramer at the dimer–dimer interface. PDB entries 6d23 and 6d24 are for the free enzyme and the G6P complex of a truncated form lacking the first 37 amino acids, respectively (Ortíz *et al.*, 2019 ▸). Arg323 is critical, forming two salt bridges per subunit with Asp332 and Glu333, and also stabilizing the active enzyme conformation, being in a loop just after the structural elements of the active site (Ortíz *et al.*, 2019 ▸). Only in the N-terminal domain of the *T. cruzi* G6PD are there two disulfide bonds joining subunits in the dimer. These are involved in the redox regulation of the enzyme, which is inactivated by reducing agents, similarly to cyanobacterial and chloroplast G6PDs (Wenderoth *et al.*, 1997 ▸; Wendt *et al.*, 1999 ▸; Igoillo-Esteve & Cazzulo, 2006 ▸; Gupta *et al.*, 2011 ▸; Ortíz *et al.*, 2019 ▸).

**Figure 11:**
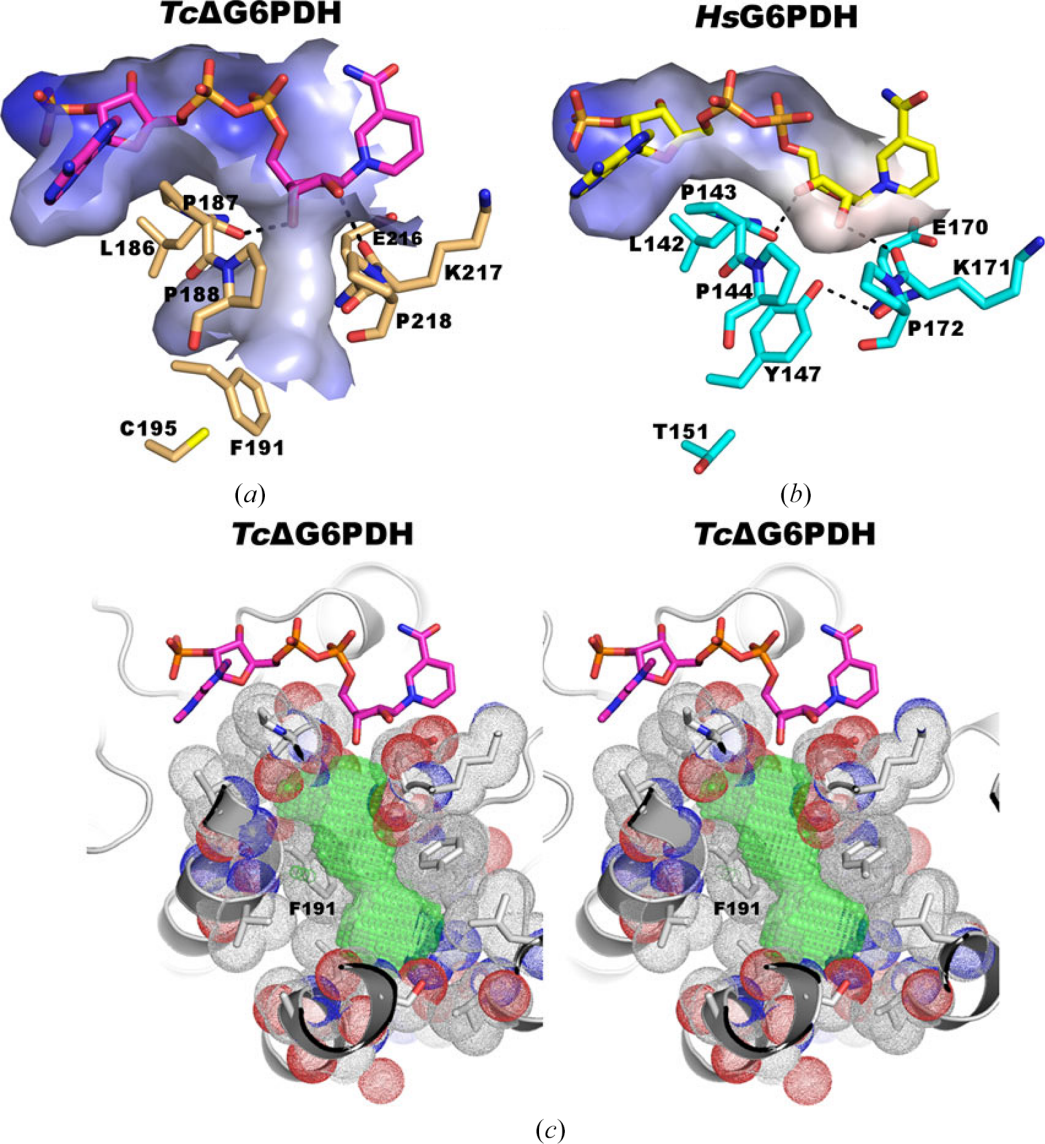
Comparison of *Tc*ΔG6PDH (*a*) and human G6PG (*Hs*G6PDH) (*b*) shows the entry of a side cavity in the parasite enzyme, close to the nicotinamide riboside, that is not present in the human enzyme. (*c*) Stereo diagram showing a section of the cavity observed in the Rossmann-like domain of *Tc*ΔG6PDH. Residues defining the surface of this cavity are mainly hydrophobic. Probes (green) were added using *KVFINDER* and were used to estimate a volume of about 220 Å^3^ (PDB entry 5aq1) [reproduced from Mercaldi *et al.* (2016 ▸) with the permission of John Wiley & Sons].

The structure of the ternary complex of a truncated *T. cruzi* G6PD, lacking 57 N-terminal and ten C-terminal residues (*Tc*ΔG6PDH), with G6P and NADPH (PDB entry 5aq1) revealed the presence of a cavity near the catalytic NADPH binding site which is not present in the human enzyme. This difference, which is exploitable in the search for parasite-specific inhibitors, arises because in *T. cruzi* G6PD a phenyl­alanine (Phe191) replaces a tyrosine (Tyr147) in human G6PD, allowing a completely different orientation far from the coenzyme binding site (Fig. 11 ▸; Mercaldi *et al.*, 2016 ▸).

**Figure 12:**
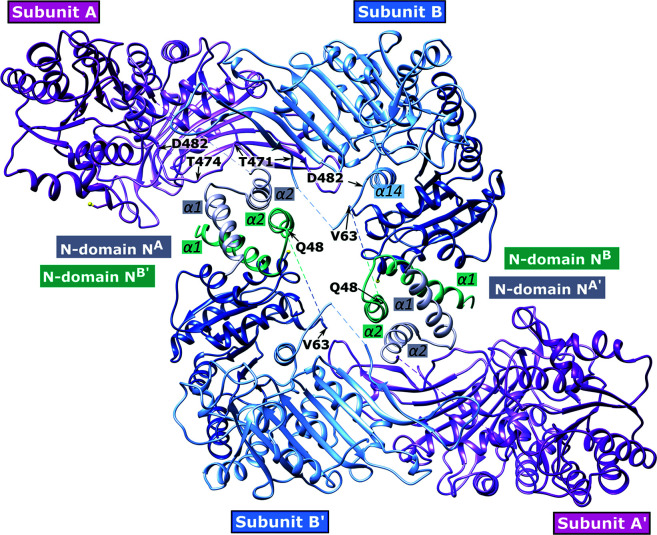
The *L. donovani* G6PD tetramer [PDB entry 7zht; reproduced from Berneburg, Ralhfs *et al.* (2022 ▸) according to https://creativecommons.org/licenses/by/4.0/].

While the *T. cruzi* and *T. brucei* G6PDs are inhibited by DHEA and epiandrosterone (EA), the *Leishmania* enzyme is insensitive to them (Cordeiro *et al.*, 2009 ▸; Cordeiro & Thiemann, 2010 ▸; Gupta *et al.*, 2011 ▸). In fact, the tetramer of *L. donovani* G6PD (PDB entry 7zht) is completely different, since the N-terminal domains form the dimer–dimer interface (face-to-face orientation; Fig. 12 ▸), whereas the other crystallized G6PDs, including those from human, LM and *T. cruzi*, present a back-to-back orientation of the two dimers (Berneburg, Rahlfs *et al.*, 2022 ▸; Fig. 8 ▸). That is, in *Leishmania* G6PD the two helical N-terminal domains are revealed to be essential for tetramerization. Also, this domain rotates after substrate binding, leading to both a decreased angle between the ‘Rossmann-like’ domain and the β+α domain and to an important shift of the active-site residues (PDB entry 7zhv; Berneburg, Rahlfs *et al.*, 2022 ▸).

## 5. Bifunctional G6PDs

**Figure 13:**
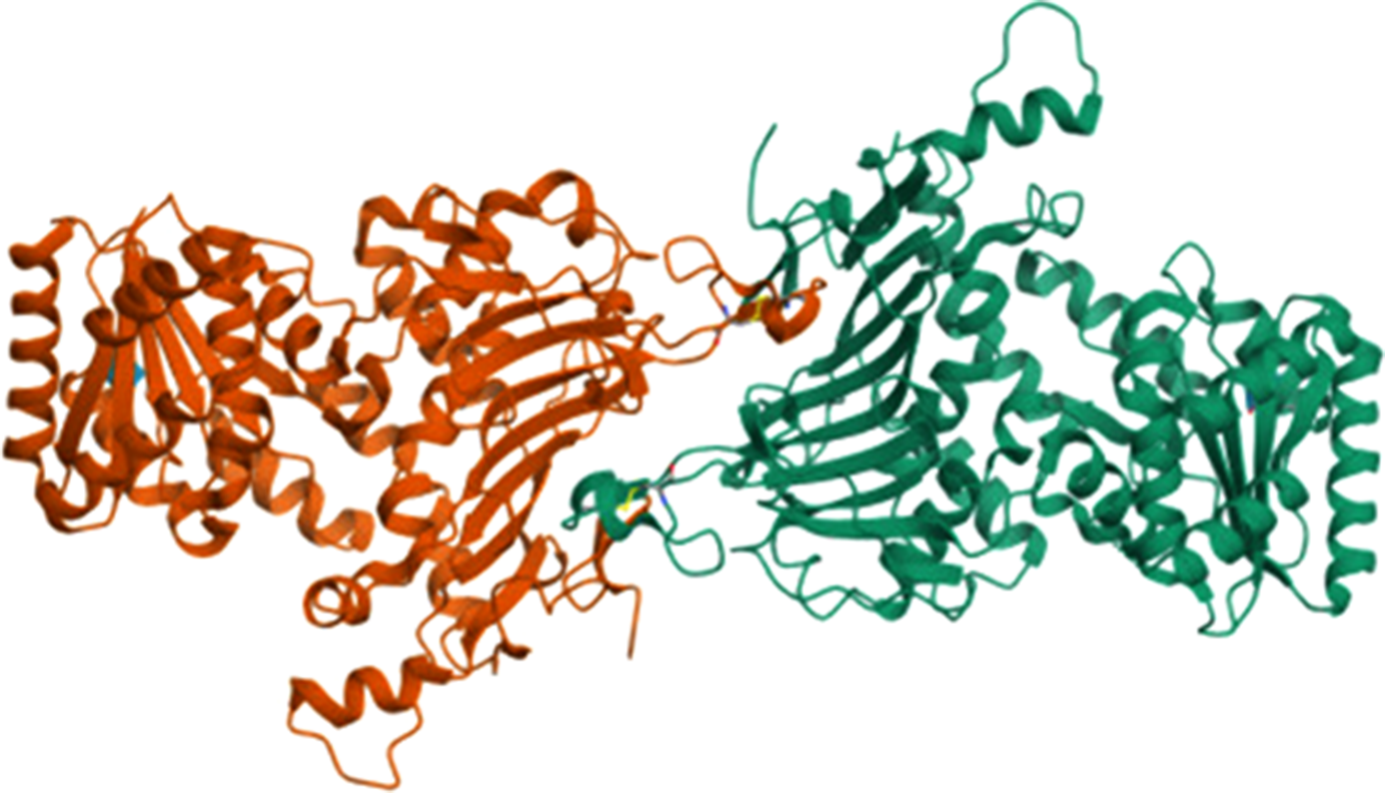
The dimeric hexose-6-phosphate dehydrogenase (H6PD) model (Su *et al.*, 2022 ▸; PDB entry 8em2).

In vertebrates, the microsomial H6PD is believed to have been derived from duplication of G6PD through sequence divergence and gene fusion (Stover *et al.*, 2011 ▸). In fact, it is a bifunctional enzyme with 6PGL activity in the C-terminus that has been shown to be important in providing NADPH in the endoplasmic reticulum (ER), which is necessary for glucocorticoid reduction and neutralization of oxidative stress, and the fine tuning of the supply of which seems to be involved in control of ER activity (Marcolongo *et al.*, 2011 ▸; Gansemer & Rutkowski, 2022 ▸). High-resolution cryo-EM recently showed a dimeric structure (Fig. 13 ▸; PDB entry 8em2) with an extended flexible loop from the β-domain of each subunit protruding into the β-domain of the other subunit (Su *et al.*, 2022 ▸). The subunit encompasses an αβ domain with six α-helices and six β-strands, an all-α-helical domain with six helices and an all-β-stranded domain with 12 β-strands (Su *et al.*, 2022 ▸).

The other fusions between G6PD and 6PGL that occur are in the microorganisms *Giardia lamblia*, *Trichomonas vaginalis* and *Plasmodium* (Morales-Luna *et al.*, 2024 ▸), although 6PGL is at the N-terminus in *Plasmodium* (Clarke *et al.*, 2001 ▸, 2003 ▸). For *G. lamblia* and *T. vaginalis* it was shown that there is a high-affinity site for NADP^+^s, as in human G6PD (Morales-Luna *et al.*, 2024 ▸). This site is between the C-terminal part of the G6PD subdomain and the 6PGL module, and coenzyme binding there increases the stability of the enzyme, especially the 6PGL part (Morales-Luna *et al.*, 2024 ▸). The 6PGL module has a similar structure to other 6PGLs, with many helices, mainly of the α-type, surrounding several β-sheets (Delarue *et al.*, 2007 ▸). Research to find selective inhibitors of parasite bifunctional G6PDs, compared with mammal G6PD, is very active, and potential antimalarial drugs with nanomolar activity have been developed (Allen *et al.*, 2015 ▸; Berneburg, Peddibhotla *et al.*, 2022 ▸).

## 6. Bacterial G6PD and FGD

**Figure 14:**
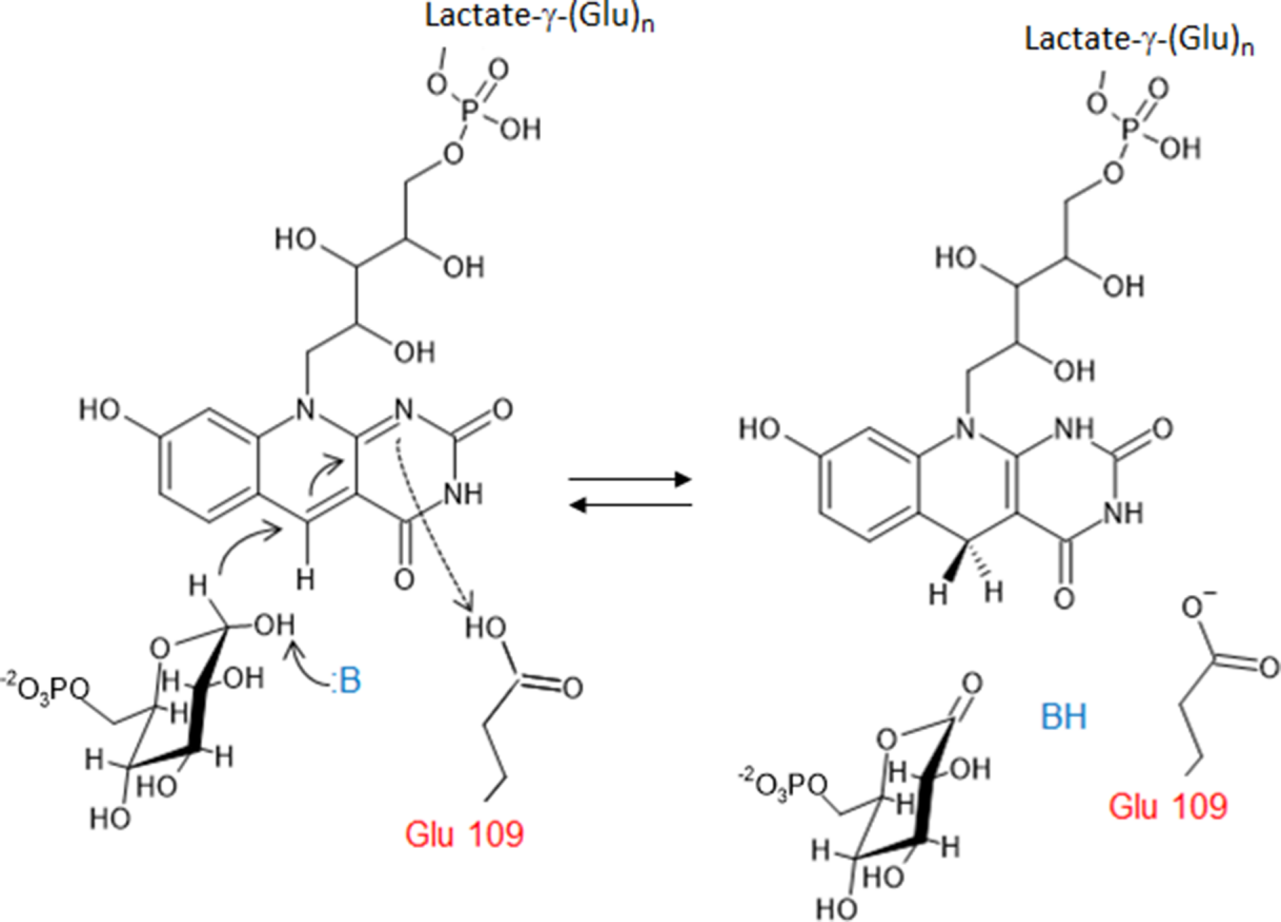
Proposed mechanism of the reaction catalyzed by FGD. An unknown base (:B) takes a proton from G6P, allowing hydride transfer to the F_420_ cofactor and oxidation of glucose 6-phosphate to 6-phosphogluconolactone; Glu109 then releases a proton to form F_420_H_2_ (for details, see Oyugi *et al.*, 2016 ▸).

**Figure 15:**
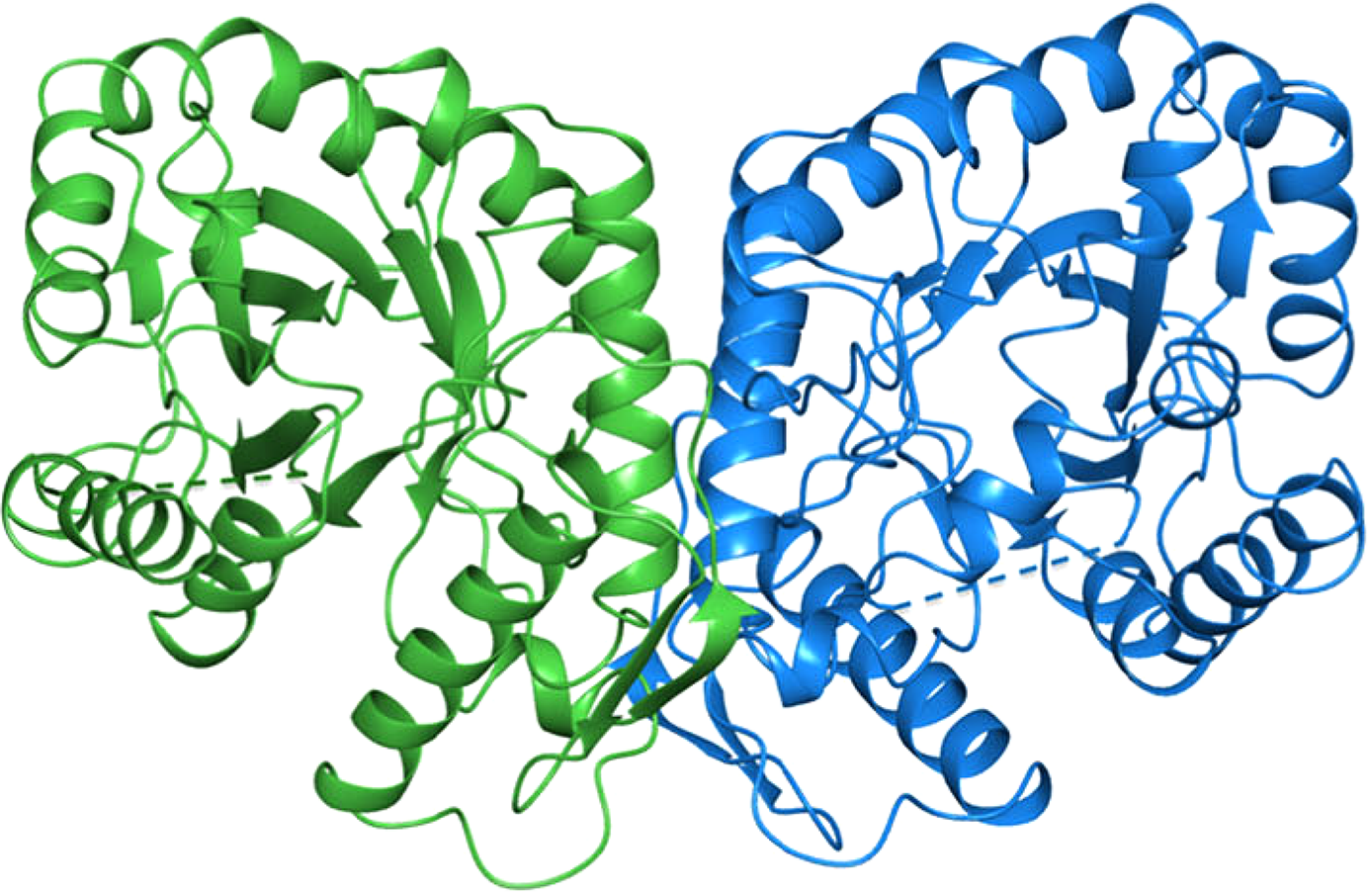
Ribbon diagram of the *Rhodococcus jostii* FGD1 dimer [PDB entry 5lxe; reproduced from Nguyen *et al.* (2017 ▸) according to https://creativecommons.org/licenses/by/4.0/].

G6PD is an ubiquitous enzyme. The solved structures in this category, including the *L. mesenteroides* and *Mycobacterium avium* G6PDs, show a similar overall structure to the other two-domain G6PDs (Rowland *et al.*, 1994 ▸; Vu *et al.*, 2021 ▸). FGD was first identified in *Mycobacterium* in 1996, using the oxidized form of F_420_ to convert G6P to 6-phosphogluconolactone, thus producing the reduced F_420_H_2_ (Fig. 14 ▸). There was no significant homology to NADP-dependent G6PD (Purwantini & Daniels, 1998 ▸). FGD belongs to an F_420_-dependent enzyme subgroup within the luciferase-like hydride transferase family (Nguyen *et al.*, 2017 ▸). F_420_ is a hydride-transfer cofactor that is widely distributed in archaea and in actinomycetes and is found in cyanobacteria (Purwantini & Daniels, 1998 ▸; Mehta *et al.*, 2015 ▸). The nitrogen in the F_420_ central ring provides a d-ribitylphospho-l-lactate elongated with several glutamate residues (from two to seven, depending on the organism; Fig. 14 ▸; Ney *et al.*, 2017 ▸). The redox potential of F_420_ is lower than that of NAD(P)^+^; thus, it is useful to reduce NADP^+^ itself and other compounds. It is essential for protecting mycobacteria against a hostile environment such as anaerobic conditions, oxidative or nitrosative stress or the presence of antimicrobial compounds, and hence is critical in the development of resistance (Purwantini & Daniels, 1996 ▸; Jacobson & Walsh, 1984 ▸; Hasan *et al.*, 2010 ▸; Jirapanjawat *et al.*, 2016 ▸; Nguyen *et al.*, 2017 ▸). While F_420_ is involved in the oxidation of energy sources such as H_2_ and formate in archaea, in *Streptomyces* spp. F_420_ participates in the biosynthesis of antibiotics (Ney *et al.*, 2017 ▸; Wang *et al.*, 2013 ▸). The cofactor also participates in the biodegradation of picrate and aflatoxins (Heiss *et al.*, 2002 ▸; Taylor *et al.*, 2010 ▸). *Rhodococcus jostii* FGD1 produced in an *M. smegmatis* culture has been proposed to possibly be useful for potential biotechnological applications of F_420_ (Fig. 15 ▸; Nguyen *et al.*, 2017 ▸).

**Figure 16:**
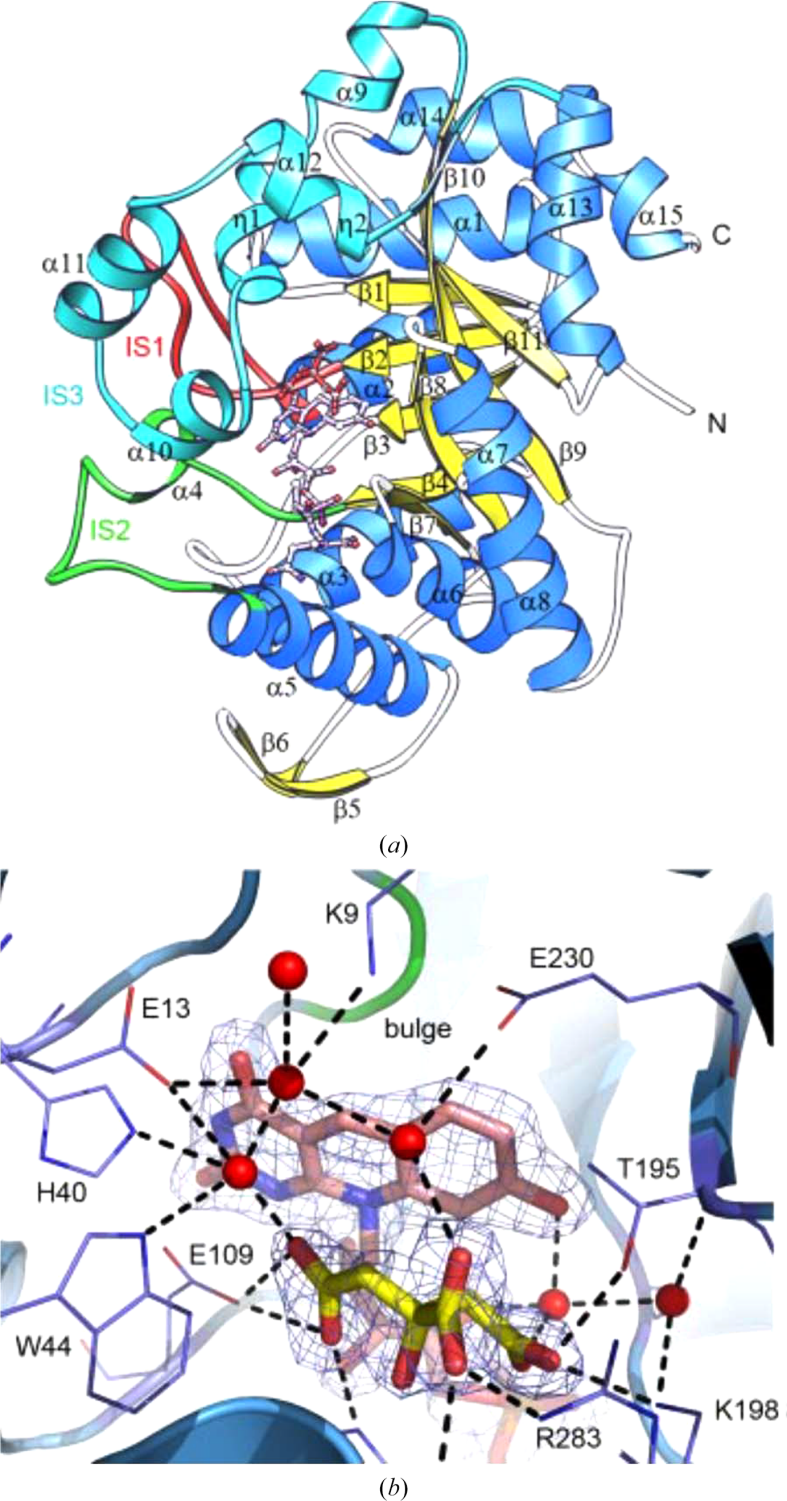
(*a*) Ribbon diagram of the *M. tubercolosis* FGD1 monomer with bound F420 and a citrate (red) in ball-and-stick representation (PDB entry 3b4y). IS are insertion sequences in the core barrel that are proposed to cap the active site. (*b*) View of the active site; water molecules are shown as red spheres (reproduced from Bashiri *et al.*, 2008 ▸).

The first crystallized FGD was from *M. tuberculosis*: a 78 kDa homodimer with one F_420_ molecule per subunit (Bashiri *et al.*, 2008 ▸). The monomer is depicted in Fig. 16 ▸(*a*), based on PDB entry 3b4y, and shows the complex with F_420_ and the substrate-competitive inhibitor citrate. The monomer is comprised of an (α/β)_8_ TIM barrel with the enzyme active site at the C-terminal end (Bashiri *et al.*, 2008 ▸). The isoalloxazine ring of F_420_ is tightly enclosed in a bulge of strand β3 containing a nonprolyl *cis*-peptide bond between a serine and a valine. It has a butterfly bend of 162° that has been proposed to make the hydride-acceptor C5 atom of isoalloxazine more reactive (Bashiri *et al.*, 2008 ▸; Oyugi *et al.*, 2018 ▸). Mechanistic studies suggest that F_420_ binds before G6P, with this then stacking against the F_420_ ring, with the phosphate in a pocket formed by two lysines and an arginine (Fig. 16 ▸*b*; Bashiri *et al.*, 2008 ▸). Glu109 is the active-site acid protonating the cofactor at position N1, while the base which abstracts hydrogen from the C1-OH of G6P, thus facilitating hydride transfer from C1 of G6P to C5 of F_420_, does not seem to be His40 (Figs. 14 ▸ and 16 ▸*b*; Oyugi *et al.*, 2016 ▸). This histidine, together with Glu109, forms a pocket binding the F_420_ pyrimidine ring. Trp44 is essential to stabilize enzyme-reaction intermediates and to anchor F_420_, forming a hydrophobic wall of the pocket (Fig. 16 ▸*b*; Oyugi *et al.*, 2016 ▸; Bashiri *et al.*, 2008 ▸).

## 7. Conclusions

Complementary to the X-ray crystallographic and electron cryo-microscopy structures, there have been a diversity of biochemical studies, including those on reaction mechanisms, regulation, inhibitors, natural and artificial mutants, and post-translational modifications. There is also a strong medical interest in this G6PD family. Research on the enzyme is aiming at finding a cure for parasitic diseases such as malaria and trypanosomiasis. In addition, there is research on the role of G6PD in cancer cell metabolism. On the other hand, in the wider family, FGD is considered as a target against *M. tuberculosis* and possesses a high biotechnological potential in biocatalysis and bioremediation. Overall, we hope that this topical review will stimulate a continuing suite of studies on the G6PD family and the wider family of enzymes. In addition, we hope that it will inform all researchers involved of the wide range of research going on and provide them with a snapshot of the 3D structures of the various types of G6PDs in the PDB.

## Figures and Tables

**Table 1:** Crystallographic structures cited in this article

PDB code; name; organism; reference	Crystallization details	Resolution (Å); space group; No. of protein molecules in AU†; *R*, *R*_free_ (%)	The highest ± difference Fourier (*F*_o_ − *F*_c_) electron-density peak (viewed in *Coot*) and any specific comments therefrom†	PDB validation assessment (clashscore; specific comments of interest based on the PDB report‡)
1dpg; G6PD; *L. mesenteroides*; Rowland *et al.* (1994 ▸)	Not in PDB file. See Rowland *et al.* (1994 ▸) for details; also Adams *et al.* (1983 ▸).	2.0 Å; *P*3_2_21; chains *A* and *B*; 20.6, 25.7	No reflection data are available	8; Ramachandran outliers 0%; side-chain outliers 7.1%
1qki†§; G6PD (variant Canton R459L) complexed with structural NADP^+^; *H. sapiens*; Au *et al.* (2000 ▸)	Hanging-drop vapour diffusion. 1 + 1 µl drops in the well. 0.1 *M* sodium citrate, 0.05 *M* glycolic acid pH 5.8. Protein concentration 10 mg ml^−1^.	3 Å; *P*2_1_2_1_2_1_; 8 chains; 2 tetramers; 24.7, 29.4	49 peaks above 5σ; top peak 7.4σ; not clear how to model peaks 1–9 (maybe Fourier series-termination effects); of the top 10 peaks, peak 10 (6.1σ) is likely to be a glycerol	11; Ramachandran outliers 2.3%; side-chain outliers 7.4%; RSRZ 1.3%. 〈*I*/σ(*I*)〉 = 1.21 at 2.99 Å, *i.e.* these are relatively weak intensity data defining the ‘edge’ of the X-ray diffraction pattern; there are 20 amino acids at the N-termini which are not modelled; the eight NADP molecules fit their electron-density omit maps well. *PDB-REDO*, according to its bound water validation criteria, removed about 75% of the depositors’ bound waters.
2bh9; deletion variant of G6PD complexed with structural and coenzyme NADP^+^; *H. sapiens*; Kotaka *et al.* (2005 ▸)	Hanging-drop vapour diffusion. 1 + 1 µl drops in the well. 0.1 *M* Tris–HCl pH 7.5–8.2, 10–16% PEG 4000. Protein concentration 5 mg ml^−1^.	2.50 Å; *F*222; chain *A*; 19.6, 29.6	No reflection data are available	27; Ramachandran outliers 0.6%; side-chain outliers 14.5%. The *R*_free_/*R* factor gap of 10% is large and indicative of overfitting of the model.
2bhl†¶; G6PD (deletion variant) complexed with glucose 6-phosphate; *H. sapiens*; Kotaka *et al.* (2005 ▸)	0.1 *M* Tris pH 8.5, 0.2 *M* MgCl_2_, 12% PEG 4000, 5% glycerol pH 8.50	2.9 Å; *C*222_1_; chains *A* and *B*; 21.2, 26.1	30 peaks above 5σ; top peak 7.6σ; first ten peaks possibly bound waters; peak 11 (6.0σ) possibly a side-chain adjustment at Asn414	30; Ramachandran outliers 1.5%; side-chain outliers 13.7%; RSRZ 4.5%. Electron density on each G6P looks good.
7sei¶; G6PD (K403Q); *H. sapiens*; Garcia *et al.* (2022 ▸)††	PEG 8K, vapor diffusion, 295 K	3.65 Å; *P*4_1_2_1_2; chain *A*; 19.1, 23.5	23 peaks above 5σ; top peak 12.4σ, which is a lengthy unmodelled ‘blob’. 28 unmodelled blobs were found in *Coot*.	12; Ramachandran outliers 3.1%; side-chain outliers 7.8%; RSRZ 0%. 17% of the residues were present in the sample but not in the PDB model.
6e08¶; G6PD in complex with structural NADP^+^; *H. sapiens*; Au *et al.* (2000 ▸)‡‡	20%(*w*/*v*) PEG 3350, 0.2 *M* potassium formate pH 7.3, vapor diffusion, sitting drop, 293 K	1.9 Å; *F*222; chain *L*; 17.2, 21.3	19 peaks above 5σ; top peak 7.45σ which looks like a split occupancy possibility on the Cys232 side chain; peak 2 at 6.05σ is possibly a bound water	3; Ramachandran outliers 0%; side-chain outliers 1.2%; RSRZ 1.9%
6d23†¶; G6PD (apo form); *T. cruzi*; Ortíz *et al.* (2019 ▸)	4 µl 20 mg ml^−1^ protein (in 20 m*M* Tris pH 8.0, 50 m*M* NaCl, 0.5 m*M* MgCl_2_) + 4 µl 6% PEG 400, 1.6 *M* ammonium sulfate, 0.1 *M* HEPES pH 7.5, vapor diffusion, hanging drop, 293 K	2.85 Å; *P*2_1_; chains *A*, *B*, *C* and *D*; 20.4, 24.9	17 peaks above 5σ; top peak 7σ; not clear how to model top three peaks above 6σ	5; Ramachandran outliers 0.3%; side-chain outliers 6.4%; RSRZ 4.8%
6d24†¶; G6PD in complex with G6P; *T. cruzi*; Ortíz *et al.* (2019 ▸)	4 µl 33 mg ml^−1^ protein (in 20 m*M* Tris pH 8.0, 50 m*M* NaCl, 0.5 m*M* MgCl_2_) + 4 µl 4% PEG 400, 1.8 *M* ammonium sulfate, 0.1 *M* HEPES buffer, 5 m*M* G6P pH 7.5, vapor diffusion, hanging drop, 293 K	3.35 Å; *P*2_1_; chains *A*, *B*, *C* and *D*; 20.5, 25.4	29 peaks above 5σ; top 12 peaks all negative with top two peaks being −11.4σ and −10.2σ; all 12 peaks appear to be misassigned as sulfates or incorrect occupancy estimates	6; Ramachandran outliers 0.9%; side-chain outliers 8.9%; RSRZ 2.6%
5aq1†¶; G6PD–G6P–NADPH ternary complex; *T. cruzi*; Mercaldi *et al.* (2016 ▸)	2 µl 10 mg ml^−1^ protein, 5 m*M* G6P, 2 m*M* NADPH in 20 m*M* Tris–HCl pH 8.0, 0.2 *M* NaCl, 5 m*M* ME. Precipitant: 1 µl 30% Jeffamine ED-2003 pH 7.0, 0.1 *M* HEPES pH 7.0; vapor diffusion, sitting drop, 293 K.	2.65 Å; *I*4_1_22; chains *A*, *B* and *C*; 20.0, 22.6	58 peaks above 5σ; top three peaks occur at Tyr443*A*/*B*/*C* (10.1σ, 8.6σ and 8.6σ) each accompanied by a planar difference electron density at 5.3σ down to 3.8σ. Tyr443 is remote from the NADP or G6P binding sites (∼20 Å) and is not mentioned in the publication.	1; Ramachandran outliers 0%; side-chain outliers 2.8%; RSRZ 2.1%. The NADP and G6P molecules fit their electron-density omit maps well. PDB report: ‘The analyses of the Patterson function revealed a significant off-origin peak that is 61.85% of the origin peak, indicating pseudo-translational symmetry’.
7zhv†¶; G6PD complexed with glucose 6-phosphate; *L. donovani*; Berneburg, Rahlfs *et al.* (2022 ▸)§§	12% PEG 3000, 200 m*M* ammonium sulfate, vapor diffusion, sitting drop, 283 K	3.3 Å; *C*2; chains *A* and *B*; 25.0, 30.9	7 peaks above 5σ; top peak −6.8σ but the problem in the model that it is signifying is unclear	11; Ramachandran outliers 0%; side-chain outliers 1.4%. 12% of the residues are present in the sample but not in the PDB model.
7zht†¶; G6PD apo form; *L. donovani*; Berneburg, Rahlfs *et al.* (2022 ▸)	18% PEG 3000, 200 m*M* ammonium chloride, vapor diffusion, sitting drop, 283 K	2.8 Å; *P*2; chains *A*, *B*, *C* and *D*; 30.4, 24.4	35 peaks above 5σ; top peak −9.0σ; not clear how to model the top peaks 1–3 (may be Fourier series-termination effects)	17; Ramachandran outliers 0.2%; side-chain outliers 6.4%; RSRZ 4.7%. 6%, 7%, 14% and 14% of chains *A*, *B*, *C* and *D*, respectively, are unmodelled. 〈*I*/σ(*I*)〉 = 1.25 at 2.81 Å, *i.e.* these are relatively weak intensity data defining the ‘edge’ of the X-ray diffraction pattern. PDB report: ‘The analyses of the Patterson function reveals a significant off-origin peak that is 29.63% of the origin peak, indicating pseudo-translational symmetry’.
3b4y†¶; FGD complexed with F_420_ and citrate; *M. tuberculosis*; Bashiri *et al.* (2008 ▸)	1.4 *M* trisodium citrate pH 6.5, vapor diffusion, sitting drop, 291 K	1.95 Å; *P*2_1_2_1_2; chains *A* and *B*; 23.4, 19.2	32 peaks above 5σ; the top two peaks of 10.3σ and 8.1σ are interestingly shaped unmodelled ‘blobs’ as defined by *Coot*	4; Ramachandran outliers 0; side-chain outliers 1.8%; RSRZ 6.0%; 〈*I*/σ(*I*)〉 = 1.4 at 1.95 Å, *i.e.* these are relatively weak intensity data defining the ‘edge’ of the X-ray diffraction pattern
5lxe†¶; F420-dependent glucose-6-phosphate dehydrogenase from *R. jostii* RHA1; Nguyen *et al.* (2017 ▸)	Ammonium sulfate, sodium acetate pH 4.6, PEG 4000, vapor diffusion, sitting drop, 293.15 K	1.45 Å; *P*2_1_2_1_2_1_; chains *A* and *B*; 18.5, 15.6	48 peaks above 5σ; top two peaks 14.7σ and 13.8σ; these 48 peaks mainly look like waters but some are unmodelled ‘blobs’ and a few side chains could be remodelled	2; Ramachandran outliers 0; side-chain outliers 1.8%; RSRZ 1.9%

† Entries with more than one subunit in the asymmetric unit (AU) had their space groups checked and confirmed using *Zanuda* (Lebedev & Isupov, 2014 ▸).

‡ The validation report from the PDB concerns the derived model and not unmodelled peaks. The *F*_o_ − *F*_c_ map was inspected in the *Coot* visualization system to describe the unmodelled peaks (Emsley *et al.*, 2010 ▸).

§ The difference-map evaluation in this table used the PDB-REDO entry for PDB entry 1qki as the deposited cif file of structure factors did not have map coefficients. The PDB-REDO file has the eight chains displayed in the *Draw Sequence View* tool in *Coot* (Emsley *et al.*, 2010 ▸), whereas PDB entry 1qki shows chains *A*, *B*, *C* and *D* even though the PDB file contains all eight chains and *Coot* displays them, *i.e.* chains *A*–*H*. This is presumably a format-interconversion problem.

¶ The difference-map evaluation in this table used the PDB-REDO entry.

†† Primary citation of related structure 7seh.

‡‡ Primary citation of related structure 6e07.

§§ Primary citation of related structures 7zht, 7zhu, 7zhw, 7zhx, 7zhy and 7zhz.

**Table 2:** G6PDH cryo-EM structures

PDB code; name; organism; reference	Resolution (Å)	Primary citation of related structures as listed in the PDB entry (these include entries with symmetry applied as well as no symmetry applied)	PDB validation assessment (clashscore; Ramachandran outliers; side-chain outliers; specific comments of interest based on the PDB report)
7sng; G6PD WT tetramer; *H. sapiens*; Wei *et al.* (2022 ▸)	2.8	7snf, 7snh, 7sni, 7toe, 7tof, 7ual, 7uc2	10; 0.2%; 0.4%; portions not modelled were the N-terminal 1–28, 424–432 and 505–523 in chains *A*, *B*, *C* and *D*
7snh; G6PD D200N tetramer bound to NADP^+^; *H. sapiens*; Wei *et al.* (2022 ▸)	2.2	7snf, 7sng, 7sni, 7toe, 7tof, 7ual, 7uc2	6; 0%; 3.2%; portions not modelled were the N-terminal 1–27, 425–432 and 512–523 in chains *A*, *B*, *C* and *D*
7sni; G6PD D200N tetramer bound to NADP^+^ and G6P; *H. sapiens*; Wei *et al.* (2022 ▸)	2.5	7snf, 7sng, 7snh, 7toe, 7tof, 7ual, 7uc2	8; 0; 0.8%; 7%; portions not modelled were the N-terminal 1–27 and 516–523 in chains *A*, *B*, *C* and *D*
7snf; G6PD WT dimer; *H. sapiens*; Wei *et al.* (2022 ▸)	3.5	7sng, 7snh, 7sni, 7toe, 7tof, 7ual, 7uc2	16; 0.1%; 0%; portions not modelled were the N-terminal 1–26, 425–432 and 505–523 in chains *A* and *B*
8em2; H6PD (GDH/6PGL endoplasmic bifunctional protein); *H. sapiens*; Su *et al.* (2022 ▸)	3.02	7uzm, 8ekw, 8eky, 8emr, 8ems, 8emt, 8ene, 8eoj, 8eor	6; 0%; 3.4%; 37% of both chains *A* and *B* not modelled
